# The contribution of service density and proximity to geographical inequalities in health care utilisation in Indonesia: A nation-wide multilevel analysis

**DOI:** 10.7189/jogh.10.020428

**Published:** 2020-12

**Authors:** Joko Mulyanto, Anton E Kunst, Dionne S Kringos

**Affiliations:** 1 Department of Public Health and Community Medicine, Faculty of Medicine, Universitas Jenderal Soedirman, Purwokerto, Indonesia; 2 Department of Public and Occupational Health, Amsterdam UMC, University of Amsterdam; and Amsterdam Public Health research institute, Amsterdam, Netherlands

## Abstract

**Background:**

Geographical inequalities in access to health care have only recently become a global health issue. Little evidence is available about their determinants. This study investigates the associations of service density and service proximity with health care utilisation in Indonesia and the parts they may play in geographic inequalities in health care use.

**Methods:**

Using data from a nationally representative survey (N = 649 625), we conducted a cross-sectional study and employed multilevel logistic regression to assess whether supply-side factors relating to service density and service proximity affect the variability of outpatient and inpatient care utilisation across 497 Indonesian districts. We used median odds ratios (MORs) to estimate the extent of geographical inequalities. Changes in the MOR values indicated the role played by the supply-side factors in the inequalities.

**Results:**

Wide variations in the density and proximity of health care services were observed between districts. Outpatient care utilisation was associated with travel costs (odds ratio (OR) = 0.82, 95% confidence interval (CI) = 0.70-0.97). Inpatient care utilisation was associated with ratios of hospital beds to district population (OR = 1.23, 95% CI = 1.05-1.43) and with travel times (OR = 0.72 95% CI = 0.61-0.86). All in all, service density and proximity provided little explanation for district-level geographic inequalities in either outpatient (MOR = 1.65, 95% CrI = 1.59-1.70 decreasing to 1.61, 95% CrI = 1.56-1.67) or inpatient care utilisation (MOR = 1.63, 95% CrI = 1.55-1.69 decreasing to 1.60 95% CrI = 1.54-1.66).

**Conclusions:**

Supply-side factors play important roles in individual health care utilisation but do not explain geographical inequalities. Variations in other factors, such as the price and responsiveness of services, may also contribute to the inequalities. Further efforts to address geographical inequalities in health care should go beyond the physical presence of health care infrastructures to target issues such as regional variations in the prices and responsiveness of services.

Geographical factors have been identified as important determinants of individuals’ access to health care [1]. A recent report showed substantial geographical variations both between and within 13 OECD countries with respect to health care access [2]. The European Commission recently identified geographical disparities as an important dimension in such unequal access [3]. The causes of geographical inequalities in high-income countries, where universal health care coverage has been achieved for decades and such disparities should be minimal, are still largely unknown. A study in five OECD countries has shown that, in addition to demographic and need factors, compositional factors such education level and employment status contribute to geographic inequalities [4]. However, a large proportion of regional health care access variation still remain unexplained, suggesting that supply-side factors such as service availability might play important roles.

In low- and middle-income countries (LMICs), studies investigating geographic inequalities in health care have focused mostly on differences in utilisation between rural and urban areas and on proximity to health care facilities [5,6]. Studies that assess geographical health care inequalities among regions have been mostly concerned with disparities in health care resources, such as unequal distributions in budget, personnel, and facilities [7-9]. In the context of Indonesia, geographical inequalities have been reported in the use of reproductive, maternal, and child-related health care among provinces [10]. Our previous study found extensive district-level inequalities in general health care utilisation, which were explained only modestly by compositional factors [11]. Studies are still lacking that specifically assess the role of supply-side factors on interregional inequalities, and particularly between smaller areas such as districts. Solid empirical evidence is needed to clarify the role of supply-side factors and help guide the development of policies to address geographical inequalities in health care use, particularly in LMICs.

Indonesia has a mixture of public and private systems of health care financing and delivery. The health care delivery system is a tiered network in which primary care facilities are the main providers as well as the entry points for accessing health care. Public primary health care centres (PHCs) provide the bulk of primary care services, particularly in rural areas and to lower-SES groups while private primary care providers are private clinics and solo physician practices [12]. In secondary care, public hospitals provide most of the services but, in cities and more urbanised regencies, private hospitals and private specialist clinics have grown rapidly. Tertiary care is usually provided by academic hospitals located only in the big Indonesian cities [13]. The large-scale expansion of NHI had produced an increase in insurance coverage of the Indonesian population from about 40% in 2013 to about 70% in 2018 [14].

Considering the features of health care system and the extent of existing geographical inequalities in health care access in Indonesia, we use Indonesian data to fill this evidence gap by assessing the contributions of supply-side factors to such inequalities. We investigated the association of variables representing *service density* – ratios of general practitioners (GPs), PHCs, nurses, and hospital beds to the district population – and *service proximity* – district-level travel costs and travel times – with health care use inequalities. Specifically, we aimed to (i) map the distribution of service density and proximity among Indonesian districts, (ii) estimate district-level associations of service density and proximity with individual health care use, and (iii) estimate contributions of district-level service density and proximity to overall geographical inequalities in health care utilisation. Our study will test the following hypotheses (i) service density and service proximity are positively associated with individual health care utilisation, (ii) service density and service proximity explain the geographical inequalities in health care use in Indonesia.

## METHODS

### Study design and data source

We performed a cross-sectional study using data from the 2013 Basic Health Research (RISKESDAS) survey, a nationally representative survey conducted by the Indonesian Ministry of Health. The survey included 649 625 adult individuals aged 18 or older from all 33 provinces and 497 districts in Indonesia. The minimum sample size for a district was around 400 individuals, with the majority of districts having sample sizes around 4000. More detailed information about RISKESDAS 2013 can be found elsewhere [15]. Data about service density at the district level (the second-level administrative area in Indonesia) were extracted from the 2013 Indonesian Health Profile published by the Ministry of Health Republic of Indonesia [16].

### Measures

The main outcome variables in our study were outpatient and inpatient health care utilisation at the individual level. Self-reported outpatient care utilisation data were based on responses to the question “Did you visit outpatient care facilities for a medical purpose during the past month?” in the RISKESDAS 2013 individual questionnaire; included were outpatient care facilities in public or private hospitals, public primary health care centres, and private physician practices. Similarly, self-reported inpatient care data were obtained from the question “Were you hospitalised in healthcare facilities for a medical purpose during the past twelve months?”; this referred to both public and private hospitals.

We included two geographical factors – region and type of district – as descriptive variables in our analysis, and both were provided by the RISKESDAS 2013 data set. We regrouped the 33 provinces (first-level administrative areas) into seven “regions” based on the major inhabited Indonesian islands and their similarity in socioeconomic and cultural background. The regions were Java, Sumatra, Bali & Nusa Tenggara, Kalimantan, Sulawesi, Maluku, and Papua. “Type of district” was judged by the degree of urbanisation of a district; districts were categorised as “cities”, “regencies with high population density”, and “regencies with low population density”. We defined the degree of urbanisation via criteria from the Central Bureau of Statistics and the population density in each district as provided by the Ministry of Interior [17].

We measured two aspects of the supply side: service density and service proximity at district levels. *Service density* was defined in terms of the ratios of health personnel and health care infrastructure to district population. Data were extracted from the 2013 Indonesian Health Profile published by the Ministry of Health [16]. We included the ratios of GPs, nurses, and public primary health care centres (PHCs) for the analysis of outpatient care use, while hospital bed and nurse ratios were used to analyse inpatient care use. We categorised the service density variables into tertiles labelled “low”, “medium”, and “high”.

For *service proximity*, we used two measures: average travel time and travel costs to health care services. Travel time was calculated using data from the RISKESDAS 2013 individual questionnaire that recorded the minutes needed to travel to the nearest primary health care facility to obtain outpatient care and to a hospital to obtain inpatient care. Averages were calculated by aggregating the individual data at district level. A similar procedure was applied for the district-level average travel costs to the nearest facilities. We adjusted the nominal value of travel costs, expressed in Indonesian rupiahs (IDR), to the geographical differences in purchasing parity, using consumer price index data from the Indonesian Central Bureau of Statistics. Travel time and travel costs were categorised into tertiles.

Several individual socioeconomic factors were used as control variables, considering their strong connections to individual health care use [18]. Level of wealth was calculated at the household level and categorised into quintiles by RISKESDAS, using an index based on possession of durable items. Educational level was based on the highest level of education attained by RISKESDAS respondents. Education was categorised into five levels based on the International Standard Classification of Education (ISCED) 2011: pre-primary, primary, lower secondary, upper secondary, and tertiary education. Health insurance status was recorded in the RISKESDAS 2013 at individual level and categorised into four groups: “uninsured”, “civil servant insurance”, “public health insurance for the poor”, and “private health insurance”.

To adjust health care utilisation by health-related need, we included demographic factors such as age and sex. To assess health condition, we included self-assessed health (SAH), classified from responses to the question “In general, how do you rate your general health status over the past month?” from the RISKESDAS individual questionnaire with answer options “good”, “moderate”, and “bad”.

### Statistical analysis

The basic individual-level characteristics of the study sample were described using frequencies and percentages. We calculated the direct age- and sex-standardised prevalence rate (SPR) of health care use for each corresponding individual characteristic (for instance wealth level). Service density and proximity at district levels were described using frequencies, percentages, medians, and minimum and maximum values. To describe the variations in service density, service proximity, and health care use among districts, we displayed those variables in maps of all districts in Indonesia using QGIS, version 3.4 (QGIS.org, Berne, Switzerland).

We used regression analysis to assess the associations of service density and proximity with individual health care utilisation and to estimate their contributions to the overall geographic inequalities. Considering the hierarchical structure of the data (individuals nested to district and districts nested to province) and that the main outcome variable was measured in nominal dichotomous scale, we applied three-level logistic regression to assess the associations of service density and proximity with individual health care utilisation and to estimate their contributions to the overall geographic inequalities. We developed three models. In the first model, we included sociodemographic factors, self-assessed health, and geographical descriptors. That model was used to provide baseline values for the overall extent of geographic inequalities in health care utilisation, expressed as median odds ratios (MORs) with 95% credible intervals (95% CrI). In the second model, we added service density variables to estimate their associations with individual health care use and their possible contribution to geographical inequalities. In the third model, we added service proximity variables to obtain a similar estimation. As logistic regression was applied to assess the associations between service density and proximity variables and individual health care use, the results were expressed as odds ratios (OR) with 95% confidence intervals (CI). The contributions of service density and proximity to geographic inequalities were indicated by the changing values of intra-class correlations (ICCs) and median odds ratios (MORs). ICC is defined as the proportion of total observed individual variation in the outcome that is associated with cluster variation. The MOR can be interpreted as the median change in individuals’ likelihood of utilising health care, were they to move from one randomly selected district or province to another randomly selected one [19]. All multilevel logistic regression analyses were conducted using the statistical package Stata SE 14.2 (StataCorp LLC, College Station TX, USA).

### Ethics review

The RISKESDAS 2013 was approved by the Health Ethics Research Commission, the National Institute of Health Research and Development, and the Ministry of Health in Indonesia. The data set contained no personal identification information linkable to respondents which make this study is categorised as being exempt of human research by National Institute of Health (NIH) and not required to have further ethics approval.

## RESULTS

Women, older age groups, and individuals who rated their health as “bad” had much higher rates of health care utilisation (**Table 1**). Higher levels of health and education were associated to a small extent with greater health care use. Having health insurance (particularly civil servant or private insurance) showed a strong association.

**Table 1:** Individual characteristics of the study sample and the corresponding utilisation of health care

		Healthcare utilisation			
	**n (%)**	**Outpatient**		**Inpatient**	
		SPR (95% CI)*	**OR (95% CI)†**	SPR (95% CI) *	**OR (95% CI)†**
**Sex:**					
Men	310 671 (47.8)	6.46 (6.37-6.45)	1.00	1.83 (1.77-1.87)	1.00
Women	338 954 (52.2)	8.85 (8.74-8.93)	1.39 (1.36-1.42)	2.21 (2.16-2.26)	1.19 (1.15-1.23)
**Age group:**					
18-30	157 101 (24.2)	5.30 (5.18-5.40)	1.00	1.61 (1.55-1.67)	1.00
31-40	159 454 (24.5)	6.53 (6.40-6.64)	1.20 (1.17-1.24)	1.61 (1.54-1.66)	0.96 (0.91-1.02)
41-50	147 271 (22.7)	8.00 (7.86-8.13)	1.44 (1.40-1.49)	1.84 (1.77-1.90)	1.01 (0.95-1.07)
51-60	103 543 (15.9)	9.68 (9.50-9.86)	1.70 (1.64-1.75)	2.47 (2.37-2.56)	1.30 (1.23-1.39)
61-70	52 278 (8.0)	11.57 (11.29-11.84)	1.89 (1.82-1.97)	3.22 (3.06-3.37)	1.57 (1.46-1.68)
>70	29 978 (4.7)	11.58 (11.21-11.94)	1.62 (1.54-1.70)	3.76 (3.53-3.97)	1.53 (1.40-1.66)
**Self-assessed health:**					
Good	497 409 (76.5)	6.13 (6.05-6.19)	1.00	1.48 (1.44-1.51)	1.00
Moderate	140 801 (21.7)	12.11 (11.92-12.28)	2.08 (2.03-2.12)	3.21 (3.11-3.30)	2.26 (2.17-2.35)
Bad	11 415 (1.8)	24.71 (23.70-25.70)	4.41 (4.20-4.62)	10.03 (9.36-10.70)	7.73 (7.21-8.29)
**Wealth level:**					
Quintile 1 (poorest)	119 737 (18.4)	7.31 (7.16-7.45)	1.00	1.14 (1.07-1.19)	1.00
Quintile 2	125 770 (19.4)	7.16 (7.01-7.29)	1.15 (1.11-1.19)	1.61 (1.54-1.68)	1.50 (1.39-1.62)
Quintile 3	131 971 (20.3)	7.65 (7.51-7.77)	1.26 (1.22-1.31)	1.98 (1.90-2.05)	1.80 (1.67-1.94)
Quintile 4	135 950 (20.9)	8.34 (8.18-8.48)	1.36 (1.31-1.41)	2.51 (2.42-2.59)	2.12 (1.97-2.29)
Quintile 5 (richest)	136 197 (21.0)	8.31 (8.16-8.46)	1.38 (1.33-1.44)	2.90 (2.81-2.99)	2.37 (2.19-2.56)
**Education level:**					
Pre-primary	134 354 (20.7)	7.90 (7.73-8.06)	1.00	1.57 (1.49-1.64)	1.00
Primary	193 807 (29.8)	7.48 (7.36-7.60)	1.16 (1.13-1.19)	1.73 (1.66-1.78)	1.17 (1.10-1.23)
Lower secondary	107 915 (16.6)	8.02 (7.82-8.21)	1.17 (1.13-1.22)	2.27 (2.15-2.38)	1.27 (1.19-1.36)
Upper secondary	163 063 (25.1)	8.58 (8.39-8.76)	1.12 (1.08-1.16)	2.71 (2.60-2.82)	1.26 (1.18-1.35)
Tertiary	50 486 (7.8)	9.37 (9.06-9.68)	1.11 (1.06-1.16)	3.41 (3.21-3.61)	1.35 (1.24-1.47)
**Health insurance status:**					
Uninsured	2 801 569 (43.1)	5.56 (5.47-5.64)	1.00	1.45 (1.40-1.49)	1.00
Civil servant insurance	59 943 (9.2)	10.42 (10.17-10.65)	1.85 (1.79-1.92)	3.69 (3.53-3.83)	1.99 (1.87-2.11)
Public insurance for the poor	273 553 (42.1)	8.93 (8.82-9.03)	1.52 (1.49-1.56)	2.01 (1.96-2.06)	1.43 (1.36-1.49)
Private health insurance	35 973 (5.6)	10.55 (10.09-11.00)	1.61 (1.54-1.69)	4.09 (3.79-4.38)	2.12 (1.98-2.28)

SPR – standardised prevalence rate, CI – confidence interval, OR – odds ratio

*Age- and sex-standardised prevalence rate with 95% confidence interval, per 100 persons (except in sex and age groups standardised for age or sex only).

†Odds ratio with 95% confidence interval adjusted for age, sex, self-assessed health, wealth, education level, health insurance, type of district, and region.

**Table 2** shows the variations in service density and service proximity among districts. In terms of service density, the variation of median GP-to-population ratio among districts can be as high as three times, with similar differentials observed in the ratios of nurses and public primary health care centres (PHCs) to district population. The ratios of hospital beds showed even greater inter-district variation, with the variation between districts can be as high as seven times. In terms of service proximity, wide variations in travel times and travel costs were observed, particularly for inpatient care.

**Table 2:** Characteristics of Indonesian districts in terms of service density and proximity

	n (%)	Median	Interquartile range	Minimum	Maximum
			**(IQR)**		
**Service density**					
GP: population ratio:*					
Low	165 (33.2)	9.7	3.95	2.5	13.0
Medium	166 (33.4)	17.8	5.43	13.1	23.6
High	166 (33.4)	32.5	16.05	23.8	113.8
**Nurse: population ratio:†**					
Low	165 (33.2)	69.3	30.80	17.6	97.6
Medium	167 (33.6)	138.2	42.80	98.8	188.5
High	165 (33.2)	270.1	121.80	188.6	758.7
**PHC: population ratio:‡**					
Low	164 (33.0)	0.83	0.28	0.37	1.15
Medium	167 (33.6)	1.58	0.56	1.16	2.17
High	166 (33.4)	3.26	1.79	2.18	21.34
**Hospital beds: population ratio:§**					
Low	165 (33.2)	28.6	41.85	0.0	53.4
Medium	167 (33.6)	75.5	20.18	53.6	106.0
High	165 (33.2)	203.1	170.35	106.1	1616.0
**Service proximity**					
**Travel time to primary care facility‖:**					
Short	165 (33.2)	12.77	2.98	2.00	14.94
Medium	166 (33.4)	17.51	2.78	14.95	21.01
Long	166 (33.4)	25.78	12.69	21.03	302.03
**Travel time to hospital:‖**					
Short	165 (33.2)	25.38	11.51	5.00	35.37
Medium	166 (33.4)	43.70	9.69	35.58	55.62
Long	166 (33.4)	80.79	53.12	55.73	490.91
**Travel costs to primary care facility:¶**					
Low	165 (33.2)	2.47	0.86	0.00	3.22
Medium	166 (33.4)	3.90	0.91	3.23	5.11
High	166 (33.4)	6.99	4.16	5.14	57.44
**Travel costs to hospital¶:**					
Low	165 (33.2)	5.52	2.50	1.62	7.99
Medium	166 (33.4)	11.12	4.60	8.01	16.87
High	166 (33.4)	41.14	51.39	17.10	3277.37

*General practitioners (GP) per 40 000 district residents.

†Nurses per 100 000 residents.

‡Public primary health care centres (PHC) per 30 000 residents.

§Beds per 100 000 residents.

**‖**In minutes.

¶In thousands of Indonesian rupiahs (IDR)

**Figure 1:**
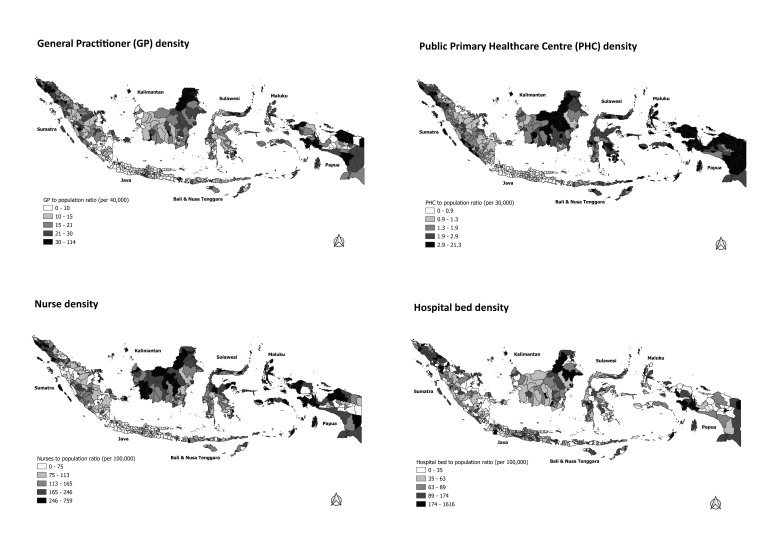
Service density by districts.

**Figure 2:**
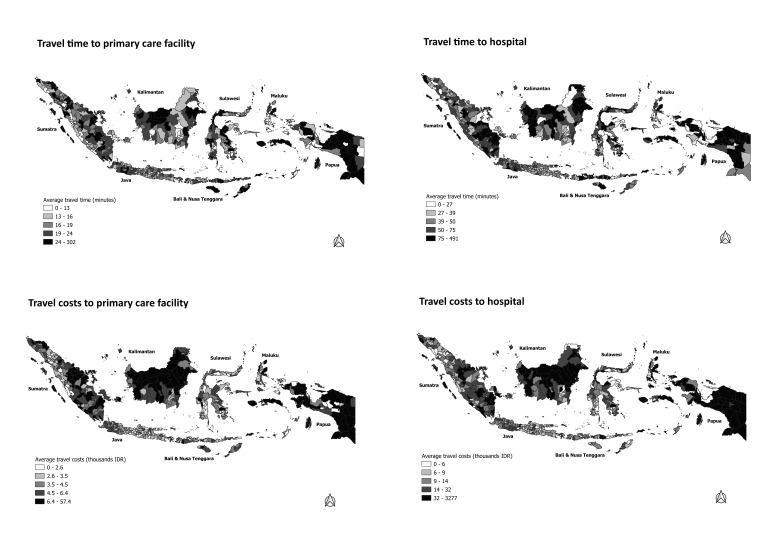
Service proximity by districts.

**Figure 3:**
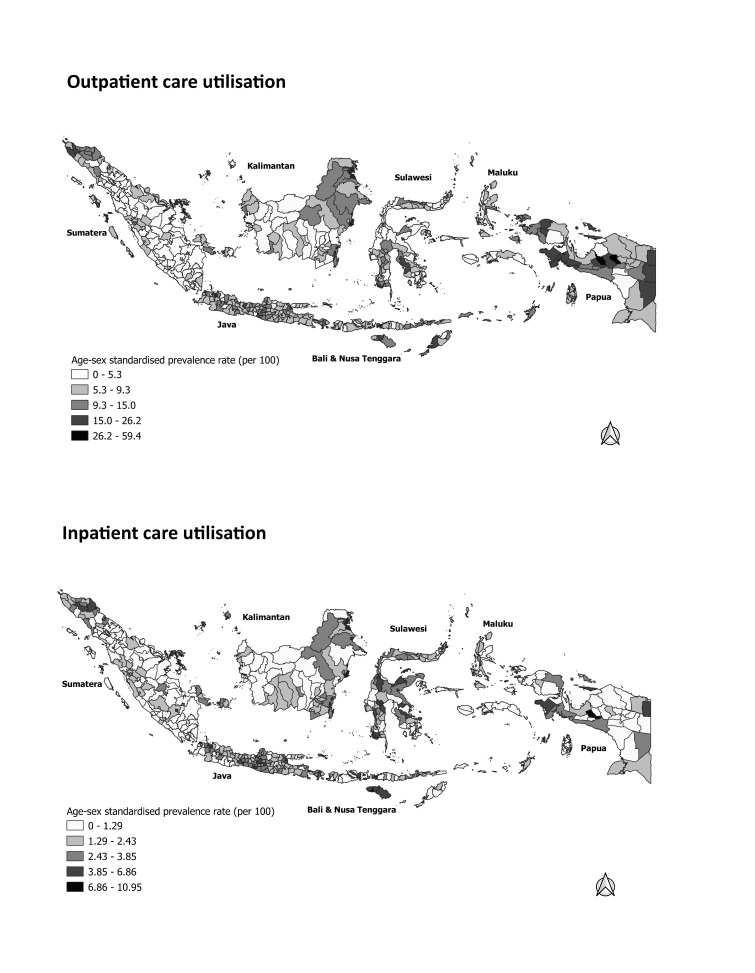
Healthcare utilisation by districts.

**Figure 1** depicts the overall geographical patterns of service density among Indonesian districts. Districts with higher population ratios of GPs, PHCs, and nurses were clustered in western Sumatra, eastern Kalimantan, and Papua. In terms of hospital beds, most districts in Java had higher ratios, while substantial numbers of districts in middle Sumatra, Maluku, and Papua had low ratios. The geographical patterns of service proximity among districts are displayed in **Figure 2**. The average travel times and costs to the nearest primary care facility or hospital were especially long and high in most districts in Sumatra, Kalimantan, and Papua, as well as in remoter districts, such as in Maluku and Nusa Tenggara. Geographical patterns of outpatient and inpatient health care utilisation are depicted in **Figure 3**. Most districts in Java and Bali had relatively high outpatient and inpatient rates. Most districts in Sumatra, Kalimantan, Maluku, had low rates of outpatient and inpatient care. The detail data of health care utilisation, service density, and service proximity for 497 district are displayed in Table S1 in the **Online Supplementary Document**.

Relationships between supply-side factors and the utilisation of individual outpatient care are analysed in **Table 3**. In terms of supply density, model 2 shows that a higher ratio of PHCs to district population was associated with greater individual uptake of outpatient care (OR = 1.22, 95% CI = 1.01-1.48). In model 3, we added service proximity variables and found that higher travel costs were associated with lower outpatient uptake (OR = 0.82, 95% CI = 0.70-0.97). The overall extent of geographic inequalities in outpatient care utilisation in Indonesia (model 1) is reflected by the median odds ratios at province level (MOR = 1.21, 95% CrI = 1.09-1.29) and at district level (MOR = 1.65, 95% CrI = 1.59-1.70). Model 2 highlights the contribution of service density to the geographic inequalities, as indicated by declining MOR values at province level (from 1.21, 95% CrI = 1.09-1.29 to 1.17, 95% CrI = 1.07-1.26) and at district level (1.65, 95% CrI = 1.59-1.70 to 1.63, 95% CrI = 1.58-1.69). Service proximity did not contribute to geographic inequalities at province level (MOR unchanged at 1.17 from model 2 to 3) but did contribute slightly at district level (MOR decreasing from 1.63, 95% CrI = 1.58-1.69 to 1.61, 95% CrI, 1.56-1.67).

**Table 3:** Associations of service density and proximity with outpatient care utilisation and their contributions to geographical inequalities

		Outpatient care utilisation			
	**n (%)**	SPR (95%CI) *	**Model 1**	**Model 2**	**Model 3**
			**OR (95% CI) †**	**OR (95% CI) ‡**	**OR (95% CI) §**
**Geographical variables**					
**Type of district:**					
Low-density regencies	210 213 (32.4)	6.60 (6.49-6.71)	1.00	1.00	1.00
High-density regencies	314 286 (48.3)	7.71 (7.62-7.80)	1.11 (0.96-1.23)	1.19 (1.02-1.39)	1.15 (0.99-1.33)
Cities	125 126 (19.3)	9.36 (9.20-9.52)	1.40 (1.17-1.67)	1.36 (1.11-1.66)	1.27 (1.04-1.55)
**Region:**					
Java	210 141 (32.3)	9.11 (8.98-9.23)	1.00	1.00	1.00
Sumatra	193 161 (29.7)	5.31 (5.20-5.41)	0.51 (0.38-0.68)	0.44 (0.34-0.57)	0.49 (0.37-0.64)
Bali & Nusa Tenggara	52 378 (8.1)	9.13 (8.88-9.38)	0.93 (0.65-1.33)	0.82 (0.59-1.13)	0.90 (0.64-1.25)
Kalimantan	62 283 (9.6)	5.96 (5.76-6.15)	0.66 (0.47-0.94)	0.54 (0.39-0.75)	0.60 (0.43-0.84)
Sulawesi	84 271 (13.0)	7.86 (7.67-8.03)	0.78 (0.57-1.06)	0.64 (0.47-0.86)	0.67 (0.49-0.91)
Maluku	19 465 (3.0)	7.35 (6.98-7.71)	0.84 (0.54-1.31)	0.67 (0.44-1.03)	0.77 (0.49-1.20)
Papua	27 926 (4.3)	13.39 (12.95-13.82)	1.90 (1.24-2.90)	1.56 (1.06-2.32)	1.72 (1.15-2.60)
**Service density**					
**GP ratio:**					
Low	252 546 (38.9)	7.12 (7.01-7.22)	-	1.00	1.00
Medium	209 932 (32.3)	7.28 (6.99-7.21)	-	0.96 (0.83-1.10)	0.94 (0.82-1.07)
High	187 147 (28.8)	8.98 (8.78-9.02)	-	1.11 (0.92-1.33)	1.05 (0.88-1.26)
**PHC ratio:**					
Low	279462 (43.0)	7.88 (7.78-7.97)	-	1.00	1.00
Medium	210 576 (32.4)	7.21 (7.09-7.31)	-	1.17 (1.00-1.36)	1.13 (0.97-1.32)
High	159 857 (24.6)	7.96 (7.82-8.09)	-	1.22 (1.01-1.48)	1.19 (0.98-1.43)
**Nurse ratio:**					
Low	260 852 (40.1)	7.07 (6.96-7.16)	-	1.00	1.00
Medium	208 876 (32.2)	8.07 (7.95-8.18)	-	1.19 (1.02-1.37)	1.18 (1.02-1.36)
High	179 897 (27.7)	8.23 (8.09-8.34)	-	1.14 (0.95-1.36)	1.13 (0.95-1.35)
**Service proximity**					
**Travel time:**					
Short	213 713 (32.9)	8.82 (8.70-8.94)	-	-	1.00
Medium	237 623 (36.6)	7.14 (7.04-7.24)	-	-	0.94 (0.83-1.07)
Long	198 289 (30.5)	7.09 (6.98-7.20)	-	-	0.88 (0.76-1.03)
**Travel costs:**					
Low	232 398 (35.8)	9.30 (9.18-9.42)	-	-	1.00
Medium	227 126 (35.0)	6.54 (6.44-6.64)	-	-	0.78 (0 · 70-0 · 90)
High	190 101 (29.2)	7.01 (6.89-7.13)	-	-	0.82 (0 · 70-0 · 97)
**ICC (95% CI)‖:**					
Province-level	-	-	0.011 (0.005-0.024)	0.007 (0.001-0.046)	0.009 (0.004-0.021)
District-level	-	-	0.088 (0.076-0.100)	0.082 (0.072-0.094)	0.080 (0.070-0.092)
**MOR (95% CrI):**¶					
Province-level	-	-	1.21 (1.09-1.29)	1.17 (1.04-1.25)	1.17 (1.07-1.26)
District-level	-	-	1.65 (1.59-1.70)	1.63 (1.58-1.69)	1.61 (1.56-1.67)

SPR – standardised prevalence rate, CI – confidence interval, OR – odds ratio, GP – general practitioner, PHC – public primary health centre, ICC – intra-class correlation, MOR – median odds ratio, CrI – credible interval

*Age and sex-standardised prevalence rate, per 100 persons. †Model 1: baseline, include geographical variables adjusted to compositional factors (wealth, educational level, health insurance), age, sex, and self-assessed health status (odds ratio with 95% confidence interval).

‡Model 2: estimating the contribution of service density variables, adjusted to all variables in model 1 (odds ratio with 95% confidence interval).

§Model 3: estimating the contribution of service proximity variables, adjusted to all variables in model 2 (odds ratio with 95% confidence interval).

**‖**Intraclass correlation with 95% confidence interval.

¶Median odds ratio with 95% credible interval.

Focusing on inpatient health care, **Table 4** analyses relationships between supply-side factors and the individual uptake of inpatient care, also in relation to the overall geographic inequalities. Model 2 shows that a higher nurse-to-population ratio was associated with higher inpatient care utilisation (OR = 1.30, 95% CI = 1.11-1.52). The addition of service proximity variables (model 3) strengthened the positive association between hospital bed ratio and inpatient care (OR = 1.23, 95% CI = 1.05-1.43), whereas long travel times correlated with lower inpatient care utilisation (OR = 0.72, 95% CI = 0.61-0.86). The overall extent of geographic inequalities in inpatient care utilisation at province and district levels in Indonesia is indicated by the respective MORs of 1.23 (95% CrI = 1.04-1.31) and 1.63 (95% CrI = 1.55-1.69) as displayed in model 1. Contributions by service density (model 2) are reflected in decreasing MOR values at province level (from 1.23, 95% CrI = 1.04-1.31 to 1.19, 95% CrI = 1.05-1.27) and district level (1.63, 95% CrI = 1.55-1.69 to 1.62, 95% CrI = 1.55-1.68). Contributions by service proximity (model 3) are shown by additional decreases at province level (1.19, 95% CrI = 1.05-1.27 to 1.16, 95% CrI = 1.00-1.23) and district level (1.62, 95% CrI = 1.55-1.68 to 1.60, 95% CrI = 1.54-1.66).

**Table 4:** Associations of service density and proximity with inpatient care utilisation and their contributions to geographical inequalities

		Inpatient care utilisation			
	**n (%)**	SPR (95%CI)*	**Model 1**	**Model 2**	**Model 3**
			**OR (95% CI)†**	**OR (95% CI)‡**	**OR (95% CI)§**
**Geographical variables**					
Type of district:					
Low-density regencies	210 213 (32.4)	1.53 (1.48-1.58)	1.00	1.00	1.00
High-density regencies	314 286 (48.3)	2.04 (1.99-2.09)	1.16 (0.99-1.35)	1.14 (0.98-1.33)	1.07 (0.92-1.25)
Cities	125 126 (19.3)	2.82 (2.72-2.91)	1.45 (1.20-1.74)	1.23 (1.01-1.52)	1.05 (0.85-1.30)
**Region:**					
Java	210 141 (32.3)	2.38 (2.31-2.44)	1.00	1.00	1.00
Sumatra	193 161 (29.7)	1.51 (1.45-1.56)	0.66 (0.49-0.89)	0.61 (0.46-0.80)	0.63 (0.49-0.80)
Bali & Nusa Tenggara	52 378 (8.1)	2.26 (2.13-2.28)	1.07 (0.73-1.56)	0.98 (0.70-1.39)	1.02 (0.74-1.40)
Kalimantan	62 283 (9.6)	1.81 (1.69-1.91)	0.88 (0.61-1.27)	0.79 (0.56-1.12)	0.85 (0.62-1.17)
Sulawesi	84 271 (13.0)	2.53 (2.42-2.63)	1.09 (0.78-1.52)	1.01 (0.74-1.37)	1.06 (0.79-1.40)
Maluku	19 465 (3.0)	1.77 (1.58-1.95)	0.86 (0.53-1.39)	0.76 (0.49-1.20)	0.89 (0.57-1.36)
Papua	27 926 (4.3)	1.78 (1.60-1.96)	1.00 (0.64-1.60)	0.87 (0.57-1.34)	0.96 (0.64-1.44)
**Service density**					
**Hospital beds ratio:**					
Low	252 546 (38.9)	1.57 (1.52-1.63)	-	1.00	1.00
Medium	209 932 (32.3)	1.88 (1.82-1.93)	-	1.07 (0.92-1.24)	1.05 (0.93-1.20)
High	187 147 (28.8)	2.62 (2.56-2.69)	-	1.10 (0.93-1.31)	1.23 (1.05-1.43)
**Nurse ratio:**					
Low	260 852 (40.1)	1.82 (1.77-1.87)	-	1.00-	1.00
Medium	208 876 (32.2)	2.09 (2.03-2.15)	-	1.09 (0.96-1.25)	1.04 (0.90-1.20)
High	179 897 (27.7)	2.27 (2.20-2.34)	-	1.30 (1.11-1.50)	1.08 (0.91-1.27)
**Service proximity**					
**Travel time:**					
Short	213 713 (32.9)	2.64 (2.57-2.70)	-	-	1.00
Medium	237 623 (36.6)	1.95 (1.89-2.00)	-	-	0.92 (0.80-1.06)
Long	198 289 (30.5)	1.42 (1.36-1.47)	-	-	0.72 (0.61-0.86)
**Travel costs:**					
Low	232 398 (35.8)	2.57 (2.50-2.63)	-	-	1.00
Medium	227 126 (35.0)	1.97 (1.91-2.02)	-	-	0.98 (0.86-1.13)
High	190 101 (29.2)	1.41 (1.35-1.46)	-	-	0.91 (0.75-1.10)
**ICC (95% CI)‖:**					
Province-level	-	-	0.013 (0.006-0.027)	0.009 (0.003-0.022)	0.007 (0.002-0.019)
District-level	-	-	0.086 (0.073-0.101)	0.081 (0.069-0.094)	0.076 (0.065-0.088)
**MOR (95% CrI):**¶					
Province-level	-	-	1.23 (1.04-1.31)	1.19 (1.05-1.27)	1.16 (1.00-1.23)
District-level	-	-	1.63 (1.55-1.69)	1.62 (1.55-1.68)	1.60 (1.54-1.66)

SPR – standardised prevalence rate, CI – confidence interval, OR – odds ratio, CI – confidence interval, ICC – intra-class correlation, MOR – median odds ratio, CrI – credible interval

*Age and sex-standardised prevalence rate, per 100 persons.

†Model 1: baseline, include geographical variables adjusted to compositional factors (wealth, educational level, health insurance), age, sex, and self-assessed health status (odds ratio with 95% confidence interval).

‡Model 2: estimating the contribution of service density variables, adjusted to all variables in model 1 (odds ratio with 95% confidence interval).

§Model 3: estimating the contribution of service proximity variables, adjusted to all variables in model 2 (odds ratio with 95% confidence interval).

**‖**Intraclass correlation with 95% confidence interval.

¶Median odds ratio with 95% credible interval.

**Table 5** and **Table 6** further analyse the associations between supply-side factors and individual health care utilisation, applying stratification by type of district (cities or regencies with high or low population densities). For outpatient care, higher service density generally tended to correspond to higher service use in regency districts. In all types of districts, service proximity tended to associate with higher service use. For inpatient care, hospital bed ratio was associated with higher service uptake regardless of district type, and service proximity tended to correspond with higher service use in all types of districts.

**Table 5:** Associations of service density and proximity with outpatient care utilisation, by type of district

	Regency of low population density		Regency of high population density		Cities	
	SPR (95% CI)*	**OR (95% CI) †**	SPR (95% CI)*	**OR (95% CI) †**	SPR (95% CI)*	**OR (95% CI) †**
**Service density**						
**GP ratio:**						
Low	5.53 (5.33-5.71)	1.00	7.56 (7.44-7.68)	1.00	12.58 (11.58-13-57)	1.00
Medium	6.03 (5.88-6.18)	0.94 (0.73-1.21)	7.93 (7.75-8.12)	0.95 (0.80-1.13)	8.89 (8.47-9.07)	0.63 (0.38-1.06)
High	7.86 (7.65-8.07)	1.07 (0.79-1.46)	9.62 (9.32-9.91)	1.17 (0.89-1.53)	9.21 (9.11-9.49)	0.79 (0.46-1.37)
**PHC ratio:**						
Low	3.21 (2.93-3.50)	1.00	7.78 (7.66-7.90)	1.00	9.57 (9.36-9.78)	1.00
Medium	5.51 (5.35-5.66)	1.17 (0.71-1.92)	7.97 (7.79-8.16)	1.17 (0.93-1.48)	8.75 (8.48-9.01)	0.98 (0.81-1.20)
High	7.57 (7.42-7.73)	1.14 (0.67-1.95)	8.35 (8.08-8.62)	1.24 (0.95-1.63)	9.46 (8.81-10.10)	1.01 (0.72-1.43)
**Nurse ratio:**						
Low	5.02 (4.83-5.22)	1.00	7.44 (7.33-7.55)	1.00	10.38 (9.87-10.89)	1.00
Medium	6.73 (6.56-6.89)	1.23 (0.92-1.61)	8.76 (8.57-8.95)	1.22 (1.01-1.47)	9.31 (9.03-9.59)	1.05 (0.72-1.53)
High	6.99 (6.68-7.17)	1.14 (0.82-1.58)	8.62 (8.32-8.92)	1.23 (0.93-1.62)	9.04 (8.83-9.25)	1.08 (0.72-1.63)
**Service proximity**						
**Travel time:**						
Short	6.47 (6.24-6.69)	1.00	9.06 (8.87-9.25)	1.00	9.82 (9.63-10.02)	1.00
Medium	5.86 (5.69-6.04)	0.94 (0.72-1.21)	7.52 (7.38-7.65)	0.97 (0.82-1.17)	8.90 (8.56-9.24)	1.08 (0.84-1.38)
Long	6.85 (6.69-7.01)	0.86 (0.67-1.11)	7.41 (7.24-7.58)	0.98 (0.79-1.22)	6.27 (5.85-6.70)	0.71 (0.51-1.01)
**Travel costs:**						
Low	7.01 (6.72-7.31)	1.00	9.20 (9.05-9.35)	1.00	10.75 (10.52-10.97)	1.00
Medium	5.57 (5.39-5.74)	0.74 (0.54-1 · 01)	6.78 (6.63-6.91)	0.78 (0.66.0.93)	7.65 (7.38-7.92)	0.89 (0.72-1.11)
High	6.81 (6.66-6.96)	0.81 (0.60-1 · 10)	7.28 (7.06-7.50)	0.88 (0.70-1.11)	6.86 (6.50-7.23)	0.85 (0.59-1.22)
**ICC (95% CI)‡:**						
Province-level	-	0.010 (0.002-0.040)	-	0.002 (0.000-0.164)	-	0.000 (0.000-1.000)
District-level	-	0.110 (0.091-0.134)	-	0.058 (0.047-0.071)	-	0.041 (0.030-0.057)
**MOR (95% CrI):§**						
Province-level	-	1.20 (1.00-1.33)	-	1.08 (1.00-1.20)	-	1.00
District-level	-	1.78 (1.66-1.90)	-	1.52 (1.44-1.59)	-	1.41 (1.34-1.50)

SPR – standardised prevalence rate, CI – confidence interval, OR – odds ratio, CI – confidence interval, GP – general practitioner, PHC – public primary health centre, ICC – intra-class correlation, MOR – median odds ratio, CrI – credible interval

*Age and sex-standardised prevalence rate, per 100 persons.

†Odds ratio adjusted to age, sex, and self-assessed health status, population density, wealth, education level, and health insurance (with 95% confidence interval).

‡Intraclass correlation (with 95% confidence interval).

§Median odds ratio (with 95% credible interval).

**Table 6:** Associations of service density and proximity with inpatient care utilisation, by type of district

	Regency of low population density		Regency of high population density		Cities	
	SPR (95% CI)*	**OR (95% CI) †**	SPR (95% CI)*	**OR (95% CI) †**	SPR (95% CI)*	**OR (95% CI) †**
**Service density:**						
Hospital beds ratio:						
Low	1.37 (1.30-1.45)	1.00	1.68 (1.61-1.76)	1.00	2.44 (2.08-2.81)	1.00
Medium	1.48 (1.39-1.56)	1.07 (0.85-1.34)	2.13 (2.06-2.21)	1.10 (0.93-1.31)	1.58 (1.27-1.89)	0.66 (0.36-1.22)
High	1.66 (1.56-1.81)	1.14 (0.87-1.49)	2.73 (2.60-2.85)	1.35 (1.08-1.69)	2.87 (2.78-2.97)	1.41 (0.89-2.22)
Nurse ratio:						
Low	1.11 (1.01-1.20)	1.00	1.97 (1.86-1.98)	1.00	3.22 (2.92-3.52)	1.00
Medium	1.53 (1.45-1.62)	1.11 (0.84-1.47)	2.47 (2.36-2.57)	1.12 (0.91-1.36)	2.43 (2.28-2.58)	0.81 (0.57-1.17)
High	1.63 (1.54-1.72)	1.22 (0.91-1.63)	2.22 (2.06-2.37)	1.20 (0.90-1.58)	2.90 (2.77-3.02)	0.90 (0.63-1.28)
**Service proximity:**						
Travel time:						
Short	1.89 (1.76-2.02)	1.00	2.64 (2.52-2.75)	1.00	2.93 (2.83-3.03)	1.00
Medium	1.52 (1.42-1.62)	0.88 (0.65-1.20)	2.09 (2.01-2.15)	0.99 (0.82-1.20)	2.26 (2.06-2.47)	0.77 (0.58-1.03)
Long	1.30 (1.23-1.36)	0.71 (0.53-0.96)	1.58 (1.49-1.66)	0.85 (0.66-1.10)	1.15 (0.75-1.56)	0.31 (0.14-0.69)
**Travel costs:**						
Low	1.83 (1.65-2.00)	1.00	2.50 (2.41-2.59)	1.00	2.86 (2.76-2.97)	1.00
Medium	1.67 (1.57-1.76)	0.96 (0.69-1.35)	2.02 (1.95-2.09)	0.98 (0.83-1.17)	2.55 (2.36-2.74)	1.04 (0.80-1.36)
High	1.29 (1.23-1.36)	0.90 (0.63-1.30)	1.50 (1.39-1.59)	0.87 (0.68-1.12)	2.40 (1.96-2.84)	1.36 (0.71-2.57)
**ICC (95% CI)‡:**						
Province-level	-	0.000 (0.000-1.000)	-	0.004 (0.000-0.045)	-	0.000 (0.000-1.000)
District-level	-	0.009 (0.078-0.125)	-	0.061 (0.048-0.078)	-	0.045 (0.031-0.065)
**MOR (95% CrI)§:**						
Province-level	-	1.00	-	1.12 (1.00-1.24)	-	1.00
District-level	-	1.76 (1.63-1.90)	-	1.52 (1.44-1.61)	-	1.44 (1.34-1.55)

SPR – standardised prevalence rate, CI – confidence interval, OR – odds ratio, CI – confidence interval, ICC – intra-class correlation, MOR – median odds ratio, CrI – credible interval

*Age and sex-standardised prevalence rate, per 100 persons.

†Odds ratio adjusted to age, sex, and self-assessed health status, population density, wealth, education level, and health insurance (with 95% confidence interval).

‡Intraclass correlation (with 95% confidence interval).

§Median odds ratio (with 95% credible interval).

## DISCUSSION

Our study focused on associations between supply-side factors, chosen to reflect district-level service density and proximity, and the individual utilisation of outpatient and inpatient health care in Indonesia. We assessed whether those factors help to explain geographical inequalities in health care use. We found large district-level variations across Indonesia, both in service density and proximity and in the individual use of health care. For outpatient care, however, none of our service density variables showed associations with utilisation rates. Higher rates of inpatient care utilisation were seen in districts with higher ratios of hospital beds to the population. In relation to service proximity, higher travel costs were associated with a lower uptake of outpatient care, and longer travel times were associated with lower uptake of inpatient care. Although supply-side factors in terms of service density and proximity thus showed some associations with individual health care use, those factors provided little overall explanation for the observed district-level geographical inequalities in health care utilisation.

Using data from a nationally representative survey with a large sample size, and combining individual and district-level data, we were able to provide a unique, detailed description of the distributions of supply-side factors and the rates of health care utilisation corresponding to them. Another innovative feature of our study is the use of median odds ratios (MORs) to estimate the magnitude of geographical inequalities in health care utilisation in Indonesia. Multilevel analysis is a standard practice in addressing issues of hierarchical data structure, but MORs have rarely been included in such analyses to enable more precise quantifications of geographic inequalities in health and health care, particularly in low- and middle-income countries (LMICs). The use of MORs allows us, moreover, to assess factors that contribute to geographic inequalities, further clarifying the nature of such inequalities.

We should consider several limitations to our study. First, we used only self-assessed health (SAH) data to adjust health care utilisation to health care need, without inclusion of other possible health conditions. Our data set lacked data on the actual health status of individuals based on objective measurement. Although our data set contained data on self-reported health conditions (diseases), such data are known to lack validity and reliability for use in inequality estimations, especially in LMICs. Second, the use of population ratios of GPs and public primary health care centres (PHCs) as a proxy for service density may not fully capture the overall district-level spectrum of primary health care provision. In Indonesia, GPs are legally permitted and commonly found to have multiple practice sites [12]. We also have not included private clinics in our analysis, due to data unavailability. With the rapid expansion of private primary care providers, their share in outpatient care services has grown [20]. Third, data on travel time and travel to indicate the service proximity were collected based on respondents’ response. Although the responses have been validated by interviewers who are familiar with the local situation, we cannot fully exclude the possibility of recall bias and response inaccuracies.

Large variations in service density among districts were found in Indonesia, even within provinces. In Indonesia, the availability of health care providers within a particular district depends on many conditions, such as local socioeconomic development and local health policy. There are vast differences in regional economic development in Indonesia which lead to wide inter-district variations in terms of living standards, education levels, and physical infrastructure [21]. Districts in more favourable socioeconomic situations provide more incentives for the growth of private health care provision, as in private physician practices and private hospitals.

In public health care provision, most PHCs and public hospitals are owned by district governments. Inadequate management by district health offices after decentralisation has affected the development of public provision [22]. Moreover, in terms of health sector development, district governments do not have to be accountable to the national health ministry and to provincial governments, even when failing to meet national standards [23]. A wide array of Indonesian government district-level decentralisation measures since 2001 may have contributed to that situation. Decentralisation has frequently been discussed as a cause of health care-related geographic inequalities both in LMICs and in higher-income countries (HICs) [24].

Surprisingly, the district-level GP and PHC ratios did not associate with individual outpatient care utilisation after adjustment with service proximity variables. Although distributions of GPs and PHCs were unequal among districts, the majority of districts in Indonesia had GP and PHC ratios above the national average. It is therefore likely that the density of supply of outpatient care was adequate in most districts. A study in Switzerland also found that the association between the number of primary care providers and outpatient uptake is not linear, which to an extent diminishes such an association [25].

For inpatient care, higher service density in terms of hospital bed ratios was associated with higher rates of inpatient care. The expansion of the Indonesian government’s health insurance programme has improved people’s access to health care and increased the demand for health care services, including inpatient care [26]. Districts with higher hospital bed ratios may better meet increased demand, resulting in higher utilisation [20]. A systematic review of studies conducted in the eastern Mediterranean countries also found that when the supply of hospital beds is adequate but not excessive, inpatient care use was increased to an optimum level and hospitals were able to perform efficiently [27].

In the context of Indonesia, travel time was not associated with outpatient care use, while higher travel costs were associated with a lower use of outpatient care. Most districts had relatively short and similar travel times (less than 25 minutes) to the nearest primary care facilities, which can be considered acceptable for most residents. Given this situation, travel costs could become the main consideration for people to visit an outpatient care provider, and it may be the remaining individual-level financial barrier especially to those with insurance. Our finding is consistent with a systematic review using data from HICs that, in areas with comparable distances to outpatient facilities, travel costs are the main consideration when choosing between different types of health care in outpatient settings [28].

Contrary to outpatient care, higher travel times were associated with lower utilisation of inpatient care, while travel costs showed no association. People’s willingness to use health care will diminish when travel time to facilities becomes very long [28]. A study in England and France has shown that people of low SES with serious health conditions are less willing to make lengthy journeys to health care facilities than those with high SES [29]. In addition to the actual travel expenses, longer travel times may also entail higher opportunity costs, such as lost income, which may financially impact mostly people with a low SES. In addition, because people with serious health conditions are likely to travel with carers, carers’ opportunity costs count as well. A study in Ireland showed that these type of costs have been identified as a major issue but are generally unrecognised by the general public and policymakers [30].

Despite relatively strong association with individual health care use, service density and proximity provided little explanation for district-level geographic inequalities in health care utilisation in Indonesia. This finding implies that such geographic disparities may be more strongly attributable to other district-specific variations related to supply- or demand-side factors – such as variations in price services and service responsiveness. These two factors will be discussed in the next two paragraphs.

The price of services may substantially influence geographic inequalities in health care use in Indonesia because approximately 60% of the Indonesian population was still uninsured in 2013, and thus often, dependent on out-of-pocket payment for health care [12]. For this segment of the population, the price of services is likely a main determinant of health care use. Moreover, the prices of private health services are not government-regulated, but follow market mechanisms [31], which may lead to wide price variations among regions. In addition to that, the prices of health care services provided by district-owned facilities are determined by district governments – a further likely cause of wide inter-district variations. For instance, some districts do not apply user charges for uninsured people obtaining health care in PHCs, and other districts apply relatively affordable charges, but many districts impose rather high user charges, seeing them as a source of local government revenue [12]. A report from the World Bank showed that Thailand faced similar issues, as the local government autonomy to determine prices within health care may have led to geographical inequality in the use of health care [32].

Variations in service responsiveness between districts may also contribute to the geographical inequalities in health care use. While the physical presence of health care facilities is a basic requirement for providing services to the population, additional factors influence whether their services respond to the needs of the population. Such factors include the number and qualification levels of health personnel and the availability of supporting equipment. The numbers of staff members in public facilities who have specific qualifications, such as physicians, specialists, nurses, and midwives, vary widely between Indonesian districts, and this is likely to affect both the types, volumes, and quality of services delivered to the population [33]. Necessary medical equipment and essential medicines are also unequally distributed amongst public facilities, due to differences in local government investment and procurement procedures [23]. Studies in LMICs and HICs showed that disparities in health care resources among local governments likely affected their capability to deliver responsive health care to the local population which may lead to geographical inequalities in health care utilisation [34-36].

District variation in service responsiveness may result from, amongst other factors, variation in financial incentive systems for health personnel who work at district-owned facilities. District-owned facilities such as PHCs and public hospitals are the main health care providers in most Indonesian districts. District government has full authority to manage the financial systems of such institutions, including the financial incentives system for employees like GPs and specialists. Variations in such systems between districts have been reported [13]. A systematic review of studies from LMICs and HICs has documented that financial incentives were a major determinant of the behaviour and performance of health personnel [37].

## CONCLUSIONS

This study has demonstrated wide inter-district variation in supply-side factors in Indonesian health care. Supply-side factors in terms of service density and service proximity were associated with resident’s use of some type of health care. However, service density and proximity offered little explanation for the geographical inequalities in health care utilisation in Indonesia. This implies that other factors, such as service prices and service responsiveness also contribute to these inequalities. Physical development of health care infrastructure, aimed at expanding the capacity of services and bringing them closer to the residents, may be the initial step towards improving access and reducing geographical disparities. However, to adequately address the geographic inequalities in health care use, additional efforts are needed, for example by targeting regional variations in the price of services and in service responsiveness.

## Additional material

Online Supplementary Document
