# Supplementary material for: The contribution of service density and proximity to geographical inequalities in health care utilisation in Indonesia: A nation-wide multilevel analysis
Source: J Glob Health. 2020 Dec 6;10(2):020428. doi: 10.7189/jogh.10.020428 (PMC7719271; doi:10.7189/jogh.10.020428)
Supplement: Online Supplementary Document [file jogh-10-020428-s001.pdf]

## Table S1. Healthcare utilisation, service density, and service proximity at district level

SPR: Age-sex standardized prevalence rate per 100 persons; GP: General Practitioner to population ratio per 40,000; PHC: Public Health Center to population ratio per 30,000; HOS: Hospital bed to population ratio per 100,000; NUR: Nurse to population ratio per 100,000; TT: Mean travel time to nearest facility in minutes; TC: Mean travel costs to nearest facility in thousand IDR.

| No | Code | Province | Code | District | SPR | 95% CI | | SPR | 95% CI | | GP | PHC | HOS | NUR | TT | TC | TT | TC |
|---|---|---|---|---|---|---|---|---|---|---|---|---|---|---|---|---|---|---|
| | | | | | | | | | | | | | | | | | | |
| 1 | 11 | Aceh SR | 1101 | Simeulue | 4.41 | 3.08 | 6.1 | 0.91 | 0.38 | 1.81 | 17.70 | 3.53 | 135.40 | 418.00 | 22.41 | 15.73 | 91.83 | 49.98 |
| 2 | | | 1102 | Aceh Singkil | 5.2 | 3.78 | 6.97 | 1.13 | 0.53 | 2.1 | 39.80 | 3.06 | 163.10 | 323.40 | 18.23 | 4.63 | 43.40 | 22.81 |
| 3 | | | 1103 | Aceh Selatan | 6.96 | 5.45 | 8.75 | 2.57 | 1.67 | 3.79 | 22.10 | 3.24 | 132.90 | 176.10 | 23.67 | 3.55 | 52.85 | 4.74 |
| 4 | | | 1104 | Aceh Tenggara | 11.6 | 9.66 | 13.8 | 0.65 | 0.25 | 1.37 | 28.70 | 2.87 | 92.90 | 210.70 | 25.19 | 4.85 | 18.89 | 6.04 |
| 5 | | | 1105 | Aceh Timur | 12.67 | 10.6 | 15 | 4.38 | 3.18 | 5.88 | 24.80 | 2.06 | 78.30 | 140.20 | 27.09 | 7.14 | 41.94 | 14.17 |
| 6 | | | 1106 | Aceh Tengah | 10.04 | 8.21 | 12.2 | 4.64 | 3.44 | 6.12 | 30.30 | 2.27 | 154.80 | 264.10 | 17.57 | 4.63 | 18.34 | 8.04 |
| 7 | | | 1107 | Aceh Barat | 9.33 | 7.67 | 11.2 | 2.75 | 1.89 | 3.87 | 24.10 | 2.13 | 188.30 | 258.90 | 15.72 | 3.93 | 28.72 | 10.48 |
| 8 | | | 1108 | Aceh Besar | 6.37 | 4.99 | 9.8 | 1.58 | 0.96 | 2.45 | 26.50 | 2.27 | 39.70 | 106.20 | 28.99 | 6.41 | 89.72 | 16.87 |
| 9 | | | 1109 | Pidie | 16.18 | 14 | 18.6 | 2.1 | 1.34 | 3.13 | 18.30 | 1.95 | 134.00 | 202.40 | 14.25 | 7.17 | 50.38 | 24.09 |
| 10 | | | 1110 | Bireuen | 13.41 | 11.5 | 15.5 | 3.76 | 2.79 | 4.97 | 27.60 | 1.32 | 194.50 | 179.30 | 19.71 | 6.50 | 27.65 | 12.60 |
| 11 | | | 1111 | Aceh Utara | 12.21 | 10.4 | 14.3 | 2.13 | 1.41 | 3.08 | 17.90 | 1.67 | 19.90 | 90.50 | 26.64 | 7.91 | 69.50 | 40.30 |
| 12 | | | 1112 | Aceh Barat Daya | 6.48 | 4.91 | 8.39 | 2.05 | 1.16 | 3.33 | 30.90 | 2.94 | 123.60 | 302.20 | 8.89 | 4.02 | 23.95 | 8.87 |
| 13 | | | 1113 | Gayo Lues | 3.39 | 2.3 | 4.81 | 1.57 | 0.84 | 2.66 | 38.20 | 4.30 | 121.80 | 231.60 | 14.98 | 3.78 | 65.93 | 14.53 |
| 14 | | | 1114 | Aceh Tamiang | 8.42 | 6.86 | 10.2 | 2.96 | 2.08 | 4.08 | 21.50 | 1.58 | 86.30 | 173.10 | 27.47 | 8.07 | 45.85 | 11.16 |
| 15 | | | 1115 | Nagan Raya | 5.33 | 4 | 6.96 | 1.27 | 0.66 | 2.2 | 32.60 | 2.86 | 95.20 | 209.50 | 13.24 | 5.89 | 34.78 | 14.27 |
| 16 | | | 1116 | Aceh Jaya | 18.28 | 15.6 | 21.2 | 1.31 | 0.67 | 2.29 | 38.30 | 3.71 | 65.60 | 320.40 | 13.79 | 5.91 | 77.54 | 40.87 |
| 17 | | | 1117 | Bener Meriah | 9.61 | 7.78 | 11.7 | 2.58 | 1.68 | 3.79 | 28.00 | 2.56 | 137.50 | 187.20 | 17.98 | 3.61 | 58.13 | 9.68 |
| 18 | | | 1118 | Pidie Jaya | 9.79 | 8.03 | 11.8 | 2.14 | 1.36 | 3.18 | 31.40 | 2.36 | 39.30 | 280.00 | 21.06 | 6.27 | 40.62 | 13.76 |
| 19 | | | 1171 | Banda Aceh | 9.11 | 7.43 | 7.43 | 2.2 | 1.43 | 3.23 | 103.30 | 1.40 | 772.00 | 689.90 | 11.08 | 4.52 | 14.67 | 6.57 |
| 20 | | | 1172 | Sabang | 20.13 | 16.6 | 24.2 | 3.18 | 1.81 | 5.14 | 71.30 | 5.58 | 334.70 | 635.20 | 20.77 | 5.47 | 26.68 | 6.60 |
| 21 | | | 1173 | Langsa | 8.11 | 6.53 | 9.95 | 4.4 | 3.27 | 5.79 | 37.00 | 0.96 | 413.20 | 376.90 | 14.34 | 3.85 | 17.99 | 4.41 |
| 22 | | | 1174 | Lhokseumawe | 11.11 | 9.25 | 13.2 | 5.96 | 4.63 | 7.54 | 38.80 | 1.00 | 367.90 | 339.10 | 15.86 | 5.16 | 21.06 | 8.60 |
| 23 | | | 1175 | Subulussalam | 8.73 | 6.71 | 11.2 | 1.22 | 0.53 | 2.35 | 25.30 | 2.11 | 0.00 | 276.00 | 19.04 | 3.79 | 42.50 | 6.96 |
| 24 | 12 | Sumatera Utara | 1201 | Nias | 3.69 | 2.58 | 5.1 | 0.37 | 0.10 | 0.95 | 21.20 | 2.19 | 275.70 | 165.80 | 42.03 | 5.29 | 51.08 | 15.32 |
| 25 | | | 1202 | Mandailing Natal | 1.18 | 0.59 | 2.1 | 0.72 | 0.30 | 1.46 | 27.50 | 1.85 | 80.50 | 64.90 | 31.05 | 3.18 | 63.56 | 9.38 |
| 26 | | | 1203 | Tapanuli Selatan | 1.99 | 1.25 | 2.98 | 0.53 | 0.18 | 1.17 | 17.50 | 1.75 | 300.00 | 65.50 | 18.84 | 3.61 | 48.75 | 9.06 |
| 27 | | | 1204 | Tapanuli Tengah | 3.39 | 2.4 | 4.65 | 1.27 | 0.68 | 2.15 | 14.80 | 2.13 | 64.50 | 95.30 | 21.51 | 4.37 | 40.42 | 8.19 |
| 28 | | | 1205 | Tapanuli Utara | 3.43 | 2.52 | 4.54 | 1.10 | 0.59 | 1.85 | 22.30 | 1.96 | 574.60 | 81.40 | 11.81 | 2.70 | 27.11 | 4.44 |
| 29 | | | 1206 | Toba Samosir | 1.84 | 1.08 | 2.92 | 0.53 | 0.19 | 1.18 | 34.40 | 3.16 | 72.50 | 211.70 | 13.75 | 3.27 | 54.69 | 10.82 |
| 30 | | | 1207 | Labuhan Batu | 2.37 | 1.59 | 3.4 | 0.56 | 0.22 | 1.15 | 18.00 | 0.90 | 341.60 | 178.00 | 20.47 | 6.40 | 69.47 | 19.50 |
| 31 | | | 1208 | Asahan | 2.42 | 1.7 | 3.33 | 0.91 | 0.51 | 1.50 | 11.20 | 0.95 | 151.70 | 48.50 | 25.90 | 4.84 | 47.63 | 9.20 |
| 32 | | | 1209 | Simalungun | 3.81 | 2.97 | 4.83 | 1.09 | 0.66 | 1.69 | 14.10 | 1.20 | 166.40 | 65.80 | 17.51 | 3.17 | 28.48 | 5.46 |
| 33 | | | 1210 | Dairi | 3.43 | 2.47 | 4.64 | 1.00 | 0.51 | 1.75 | 19.50 | 1.92 | 103.80 | 128.30 | 25.76 | 3.80 | 55.73 | 9.13 |
| 34 | | | 1211 | Karo | 4.21 | 3.15 | 5.5 | 2.37 | 1.62 | 3.35 | 29.50 | 1.56 | 246.00 | 127.70 | 20.14 | 1.69 | 54.16 | 5.66 |
| 35 | | | 1212 | Deli Serdang | 6.76 | 5.67 | 8.01 | 3.60 | 2.82 | 4.53 | 14.30 | 0.55 | 181.40 | 61.80 | 14.95 | 2.22 | 30.56 | 7.06 |
| 36 | | | 1213 | Langkat | 2.02 | 1.4 | 2.82 | 0.58 | 0.28 | 1.07 | 21.40 | 0.89 | 123.80 | 102.50 | 17.88 | 3.45 | 41.01 | 7.05 |
| 37 | | | 1214 | Nias Selatan | 22.12 | 19.5 | 24.9 | 0.43 | 0.17 | 0.90 | 10.30 | 3.58 | 88.70 | 142.10 | 37.47 | 6.13 | 95.46 | 35.16 |
| 38 | | | 1215 | Humbang Hasundutan | 3.94 | 2.83 | 5.34 | 0.48 | 0.16 | 1.12 | 24.60 | 2.01 | 0.00 | 190.60 | 17.49 | 2.32 | 80.61 | 11.53 |
| 39 | | | 1216 | Pakpak Bharat | 3.88 | 2.55 | 5.66 | 1.02 | 0.41 | 2.12 | 54.50 | 5.69 | 605.00 | 355.40 | 18.30 | 2.48 | 25.00 | 2.28 |

| | | | | | | | | | | | | | | | | | | |
|---|---|---|---|---|---|---|---|---|---|---|---|---|---|---|---|---|---|---|
| 40 | | | 1217 | Samosir | 3.19 | 2.15 | 4.53 | 2.27 | 1.40 | 3.45 | 30.50 | 2.89 | 0.00 | 182.10 | 23.27 | 2.45 | 41.15 | 5.17 |
| 41 | | | 1218 | Serdang Bedagai | 2.39 | 1.66 | 3.33 | 0.91 | 0.51 | 1.50 | 15.00 | 0.97 | 297.00 | 70.70 | 18.87 | 3.39 | 40.81 | 6.22 |
| 42 | | | 1219 | Batu Bara | 2.44 | 1.64 | 3.5 | 1.00 | 0.53 | 1.71 | 12.00 | 2.19 | 90.80 | 71.00 | 16.54 | 3.54 | 61.76 | 19.23 |
| 43 | | | 1220 | Padang Lawas Utara | 2.73 | 1.81 | 3.95 | 1.00 | 0.51 | 1.77 | 22.30 | 1.00 | 0.00 | 157.10 | 29.49 | 5.56 | 78.22 | 13.04 |
| 44 | | | 1221 | Padang Lawas | 3.97 | 2.75 | 5.52 | 0.72 | 0.27 | 1.50 | 11.10 | 1.79 | 75.60 | 46.00 | 16.00 | 2.61 | 44.81 | 7.81 |
| 45 | | | 1222 | Labuhan Batu Selatan | 0.29 | 0.08 | 0.75 | 0.58 | 0.25 | 1.14 | 21.40 | 1.76 | 75.80 | 89.50 | 25.16 | 4.38 | 33.95 | 8.39 |
| 46 | | | 1223 | Labuhan Batu Utara | 2.34 | 1.54 | 3.41 | 0.25 | 0.05 | 0.73 | 14.80 | 1.68 | 15.50 | 128.00 | 32.11 | 8.09 | 54.66 | 14.65 |
| 47 | | | 1224 | Nias Utara | 6.09 | 4.6 | 7.89 | 1.06 | 0.47 | 2.03 | 9.10 | 2.49 | 0.00 | 101.10 | 37.76 | 6.34 | 130.93 | 29.96 |
| 48 | | | 1225 | Nias Barat | 1.68 | 0.99 | 2.67 | 0.36 | 0.10 | 0.92 | 15.20 | 2.82 | 0.00 | 218.20 | 23.28 | 6.26 | 130.82 | 49.69 |
| 49 | | | 1271 | Sibolga | 7.79 | 6.06 | 9.85 | 1.52 | 0.84 | 2.52 | 35.20 | 1.36 | 584.00 | 188.60 | 11.29 | 1.66 | 16.26 | 3.60 |
| 50 | | | 1272 | Tanjung Balai | 10.38 | 8.45 | 12.6 | 0.35 | 0.07 | 1.02 | 27.30 | 1.49 | 146.60 | 163.50 | 9.45 | 3.81 | 18.18 | 7.85 |
| 51 | | | 1273 | Pematang Siantar | 3.82 | 2.83 | 5.04 | 2.14 | 1.41 | 3.10 | 38.00 | 2.33 | 615.10 | 164.80 | 14.04 | 2.39 | 19.41 | 3.26 |
| 52 | | | 1274 | Tebing Tinggi | 7.97 | 6.39 | 9.83 | 3.47 | 2.45 | 4.77 | 39.00 | 1.78 | 562.20 | 198.90 | 11.02 | 4.34 | 14.87 | 5.88 |
| 53 | | | 1275 | Medan | 5.85 | 4.81 | 7.05 | 1.65 | 1.13 | 2.34 | 45.20 | 0.54 | 822.60 | 244.20 | 13.15 | 2.77 | 41.52 | 6.67 |
| 54 | | | 1276 | Binjai | 4.66 | 3.51 | 6.07 | 1.62 | 0.97 | 2.53 | 35.50 | 0.94 | 619.70 | 234.70 | 10.08 | 3.85 | 13.44 | 5.24 |
| 55 | | | 1277 | Padangsidimpuan | 2.33 | 1.52 | 3.42 | 0.60 | 0.23 | 1.24 | 20.00 | 1.35 | 385.30 | 136.80 | 11.92 | 2.78 | 25.05 | 5.88 |
| 56 | | | 1278 | Gunung Sitoli | 5.83 | 4.44 | 7.50 | 1.65 | 0.95 | 2.65 | 7.60 | 1.37 | 194.00 | 168.80 | 22.69 | 10.99 | 40.57 | 16.05 |
| 57 | 13 | Sumatera Barat | 1301 | Kepulauan Mentawai | 7.20 | 5.40 | 9.37 | 0.54 | 0.17 | 1.27 | 26.20 | 3.74 | 99.80 | 332.00 | 50.58 | 12.42 | 181.11 | 64.00 |
| 58 | | | 1302 | Pesisir Selatan | 4.26 | 3.27 | 5.45 | 1.78 | 1.17 | 2.58 | 10.90 | 1.20 | 39.40 | 72.00 | 14.06 | 2.48 | 54.34 | 8.75 |
| 59 | | | 1303 | Solok | 6.38 | 5.07 | 7.93 | 2.26 | 1.50 | 3.26 | 12.30 | 1.47 | 70.60 | 118.40 | 13.86 | 2.36 | 40.61 | 6.36 |
| 60 | | | 1304 | Sijunjung | 6.18 | 4.80 | 7.83 | 2.12 | 1.33 | 3.21 | 25.90 | 1.70 | 63.60 | 152.10 | 17.28 | 3.97 | 43.56 | 11.07 |
| 61 | | | 1305 | Tanah Datar | 7.54 | 6.16 | 9.14 | 1.92 | 1.26 | 2.81 | 19.70 | 1.94 | 52.20 | 100.30 | 12.21 | 4.35 | 26.70 | 11.75 |
| 62 | | | 1306 | Padang Pariaman | 2.94 | 2.11 | 3.99 | 1.40 | 0.85 | 2.18 | 14.60 | 1.82 | 73.70 | 58.30 | 13.23 | 2.75 | 36.39 | 7.63 |
| 63 | | | 1307 | Agam | 7.24 | 5.87 | 8.83 | 1.24 | 0.73 | 1.96 | 9.20 | 1.38 | 10.20 | 39.90 | 15.37 | 2.59 | 41.52 | 12.84 |
| 64 | | | 1308 | Lima Puluh Kota | 5.18 | 3.97 | 6.65 | 1.73 | 1.06 | 2.65 | 14.20 | 1.80 | 70.90 | 94.90 | 15.77 | 2.87 | 35.58 | 8.79 |
| 65 | | | 1309 | Pasaman | 5.09 | 3.96 | 6.43 | 1.76 | 1.13 | 2.61 | 13.10 | 1.80 | 78.70 | 62.30 | 12.79 | 3.35 | 49.31 | 10.15 |
| 66 | | | 1310 | Solok Selatan | 1.77 | 1.02 | 2.84 | 1.03 | 0.49 | 1.90 | 33.60 | 1.58 | 0.00 | 197.70 | 16.81 | 3.93 | 70.28 | 19.98 |
| 67 | | | 1311 | Dharmasraya | 1.01 | 0.51 | 1.77 | 0.91 | 0.45 | 1.64 | 26.80 | 1.94 | 63.60 | 189.20 | 28.04 | 8.69 | 96.94 | 37.96 |
| 68 | | | 1312 | Pasaman Barat | 2.80 | 1.80 | 4.10 | 1.55 | 0.90 | 2.45 | 13.30 | 1.33 | 23.20 | 97.60 | 16.66 | 3.83 | 55.62 | 15.91 |
| 69 | | | 1371 | Padang | 2.32 | 1.65 | 3.18 | 1.29 | 0.80 | 1.97 | 95.60 | 0.75 | 306.80 | 220.00 | 14.60 | 4.87 | 28.99 | 12.34 |
| 70 | | | 1372 | Kota Solok | 14.08 | 11.66 | 16.84 | 3.97 | 2.75 | 5.55 | 68.80 | 1.92 | 412.90 | 374.50 | 9.98 | 3.13 | 13.16 | 4.33 |
| 71 | | | 1373 | Sawah Lunto | 16.34 | 13.81 | 19.19 | 3.67 | 2.54 | 5.13 | 58.50 | 3.01 | 247.40 | 339.30 | 11.77 | 2.66 | 23.51 | 6.21 |
| 72 | | | 1374 | Padang Panjang | 12.76 | 10.26 | 15.69 | 3.48 | 2.24 | 5.14 | 54.60 | 2.43 | 416.60 | 390.30 | 8.89 | 1.27 | 10.79 | 1.84 |
| 73 | | | 1375 | Bukittinggi | 7.93 | 6.30 | 9.84 | 3.22 | 2.23 | 4.49 | 65.80 | 1.79 | 663.60 | 608.90 | 10.20 | 1.97 | 14.37 | 3.21 |
| 74 | | | 1376 | Payakumbuh | 6.07 | 4.61 | 7.85 | 1.82 | 1.06 | 2.93 | 45.60 | 1.95 | 216.40 | 231.10 | 10.54 | 1.93 | 17.85 | 3.25 |
| 75 | | | 1377 | Pariaman | 5.52 | 4.22 | 7.09 | 2.80 | 1.87 | 4.01 | 73.40 | 2.53 | 87.80 | 301.90 | 14.47 | 3.76 | 18.03 | 4.95 |
| 76 | 14 | Riau | 1401 | Kuantan Singingi | 7.12 | 5.64 | 8.87 | 2.23 | 1.43 | 3.33 | 15.20 | 2.06 | 38.50 | 108.80 | 31.65 | 13.32 | 74.71 | 60.95 |
| 77 | | | 1402 | Indragiri Hulu | 4.16 | 2.99 | 5.61 | 1.24 | 0.63 | 2.17 | 13.70 | 1.29 | 29.70 | 87.20 | 17.73 | 3.71 | 29.85 | 8.61 |
| 78 | | | 1403 | Indragiri Hilir | 3.41 | 2.51 | 4.53 | 0.64 | 0.29 | 1.21 | 10.50 | 0.99 | 31.70 | 73.60 | 30.95 | 7.78 | 24.61 | 10.33 |
| 79 | | | 1404 | Pelalawan | 3.69 | 2.60 | 5.05 | 0.72 | 0.33 | 1.32 | 22.50 | 1.04 | 85.10 | 104.50 | 21.81 | 11.99 | 46.83 | 29.91 |
| 80 | | | 1405 | Siak | 2.21 | 1.43 | 3.26 | 0.79 | 0.39 | 1.42 | 17.80 | 1.04 | 26.60 | 111.90 | 14.03 | 6.93 | 69.93 | 33.23 |
| 81 | | | 1406 | Kampar | 4.00 | 2.89 | 5.37 | 0.91 | 0.47 | 1.57 | 12.00 | 1.18 | 54.40 | 64.40 | 19.21 | 8.59 | 81.43 | 36.85 |
| 82 | | | 1407 | Rokan Hulu | 2.76 | 1.85 | 3.94 | 0.84 | 0.37 | 1.60 | 12.10 | 1.16 | 54.50 | 67.30 | 16.61 | 4.51 | 36.17 | 17.89 |
| 83 | | | 1408 | Bengkalis | 7.40 | 5.96 | 9.07 | 1.01 | 0.53 | 1.74 | 19.10 | 0.58 | 91.90 | 94.20 | 21.33 | 10.02 | 40.05 | 23.14 |
| 84 | | | 1409 | Rokan Hilir | 5.75 | 4.54 | 7.17 | 2.24 | 1.51 | 3.18 | 12.60 | 0.80 | 25.50 | 93.00 | 18.54 | 6.19 | 53.74 | 24.52 |
| 85 | | | 1410 | Kepulauan Meranti | 6.28 | 4.83 | 8.02 | 0.39 | 0.10 | 0.99 | 15.80 | 1.33 | 39.00 | 75.10 | 27.54 | 9.82 | 72.17 | 99.41 |
| 86 | | | 1471 | Pekanbaru | 3.25 | 2.32 | 4.41 | 1.18 | 0.61 | 2.02 | 36.40 | 0.58 | 310.70 | 218.10 | 22.47 | 8.22 | 37.75 | 15.16 |
| 87 | | | 1473 | Dumai | 3.06 | 1.98 | 4.48 | 0.91 | 0.39 | 1.76 | 29.20 | 0.93 | 127.00 | 153.10 | 16.44 | 5.38 | 27.06 | 9.95 |

| | | | | | | | | | | | | | | | | | |
|---|---|---|---|---|---|---|---|---|---|---|---|---|---|---|---|---|---|
| 88 | 15 | Jambi | 1501 | Kerinci | 7.89 | 6.35 | 9.69 | 2.20 | 1.43 | 3.22 | 19.00 | 2.13 | 54.90 | 123.60 | 11.01 | 2.35 | 52.36 | 6.62 |
| 89 | | | 1502 | Merangin | 2.91 | 2.02 | 4.05 | 0.91 | 0.44 | 1.64 | 13.60 | 1.55 | 36.40 | 126.50 | 16.27 | 6.02 | 33.06 | 14.87 |
| 90 | | | 1503 | Sarolangun | 1.19 | 0.64 | 2.02 | 0.68 | 0.30 | 1.31 | 14.70 | 1.44 | 37.50 | 143.10 | 28.23 | 7.24 | 73.13 | 17.78 |
| 91 | | | 1504 | Batang Hari | 6.92 | 5.36 | 8.76 | 2.69 | 1.82 | 3.82 | 20.70 | 1.91 | 76.20 | 212.50 | 18.21 | 4.98 | 64.61 | 20.48 |
| 92 | | | 1505 | Muaro Jambi | 1.40 | 0.81 | 2.24 | 0.36 | 0.09 | 0.90 | 21.70 | 1.43 | 66.60 | 99.10 | 23.12 | 3.50 | 50.61 | 10.49 |
| 93 | | | 1506 | Tanjung Jabung Timur | 2.88 | 2.00 | 4.00 | 0.50 | 0.18 | 1.10 | 11.00 | 2.25 | 24.30 | 105.90 | 20.63 | 4.73 | 88.58 | 30.11 |
| 94 | | | 1507 | Tanjung Jabung Barat | 4.13 | 3.10 | 5.40 | 0.94 | 0.49 | 1.65 | 18.90 | 1.56 | 28.00 | 130.40 | 17.22 | 2.98 | 84.04 | 15.48 |
| 95 | | | 1508 | Tebo | 3.13 | 2.21 | 4.29 | 0.62 | 0.27 | 1.23 | 13.10 | 1.28 | 27.70 | 84.90 | 23.83 | 6.52 | 90.70 | 25.80 |
| 96 | | | 1509 | Bungo | 4.50 | 3.43 | 5.80 | 1.62 | 1.00 | 2.49 | 12.00 | 1.61 | 108.80 | 171.90 | 13.34 | 5.28 | 37.54 | 13.62 |
| 97 | | | 1571 | Jambi | 3.99 | 2.96 | 5.24 | 0.97 | 0.49 | 1.70 | 46.50 | 1.02 | 308.60 | 277.20 | 10.77 | 3.12 | 23.97 | 8.33 |
| 98 | | | 1572 | Sungai Penuh | 12.31 | 10.24 | 14.68 | 3.85 | 2.74 | 5.25 | 13.20 | 1.98 | 0.00 | 91.40 | 11.56 | 3.45 | 16.56 | 4.86 |
| 99 | 16 | Sumatera Selatan | 1601 | Ogan Komering Ulu | 2.05 | 1.43 | 2.83 | 0.72 | 0.37 | 1.23 | 9.20 | 1.38 | 110.40 | 78.20 | 40.10 | 9.75 | 66.06 | 13.22 |
| 100 | | | 1602 | Ogan Komering Ilir | 1.37 | 0.88 | 2.04 | 0.61 | 0.30 | 1.10 | 9.50 | 1.11 | 28.60 | 95.20 | 22.04 | 9.51 | 115.38 | 64.84 |
| 101 | | | 1603 | Muara Enim | 2.22 | 1.53 | 3.09 | 1.37 | 0.84 | 2.12 | 10.30 | 0.98 | 45.00 | 70.30 | 15.41 | 4.49 | 59.65 | 19.98 |
| 102 | | | 1604 | Lahat | 1.62 | 1.01 | 2.46 | 0.45 | 0.16 | 0.97 | 13.90 | 2.34 | 66.00 | 103.50 | 18.93 | 3.55 | 54.06 | 8.08 |
| 103 | | | 1605 | Musi Rawas | 3.73 | 2.79 | 4.88 | 1.48 | 0.88 | 2.31 | 9.60 | 1.44 | 22.70 | 116.00 | 20.51 | 4.74 | 91.75 | 16.26 |
| 104 | | | 1606 | Musi Banyuasin | 0.99 | 0.58 | 1.58 | 0.36 | 0.14 | 0.75 | 15.10 | 1.29 | 48.80 | 88.90 | 27.48 | 8.39 | 79.41 | 52.06 |
| 105 | | | 1607 | Banyuasin | 1.78 | 1.19 | 2.56 | 1.07 | 0.63 | 1.70 | 10.90 | 1.08 | 9.80 | 48.40 | 19.94 | 6.34 | 76.02 | 23.91 |
| 106 | | | 1608 | Ogan Komering Ulu Slt | 2.49 | 1.63 | 3.61 | 0.90 | 0.45 | 1.59 | 7.30 | 1.67 | 19.90 | 81.60 | 23.41 | 6.80 | 108.04 | 37.61 |
| 107 | | | 1609 | Ogan Komering Ulu Tmr | 1.95 | 1.33 | 2.74 | 0.14 | 0.02 | 0.51 | 6.10 | 1.01 | 33.60 | 83.70 | 51.03 | 9.03 | 59.92 | 24.82 |
| 108 | | | 1610 | Ogan Ilir | 1.19 | 0.67 | 1.95 | 0.56 | 0.24 | 1.11 | 7.60 | 1.83 | 0.00 | 67.00 | 78.95 | 5.37 | 118.01 | 55.62 |
| 109 | | | 1611 | Empat Lawang | 1.94 | 1.28 | 2.81 | 0.36 | 0.11 | 0.85 | 8.40 | 1.01 | 25.70 | 45.10 | 14.77 | 3.32 | 47.64 | 9.91 |
| 110 | | | 1671 | Palembang | 11.24 | 9.88 | 12.72 | 3.12 | 2.43 | 3.94 | 25.00 | 0.75 | 287.90 | 190.80 | 15.36 | 2.55 | 29.24 | 5.50 |
| 111 | | | 1672 | Prabumulih | 7.01 | 5.56 | 8.73 | 4.13 | 3.04 | 5.47 | 34.50 | 1.38 | 276.10 | 293.30 | 12.91 | 3.38 | 23.54 | 8.85 |
| 112 | | | 1673 | Pagar Alam | 6.85 | 5.40 | 8.57 | 1.72 | 1.03 | 2.69 | 21.40 | 1.55 | 118.10 | 185.30 | 12.75 | 2.65 | 25.61 | 5.22 |
| 113 | | | 1674 | Lubuk Linggau | 7.61 | 6.10 | 9.37 | 2.33 | 1.52 | 3.40 | 13.40 | 1.25 | 173.60 | 144.40 | 14.93 | 3.09 | 19.53 | 4.97 |
| 114 | 17 | Bengkulu | 1701 | Bengkulu Selatan | 4.11 | 2.94 | 5.60 | 2.23 | 1.40 | 3.36 | 26.90 | 2.76 | 70.20 | 219.90 | 12.07 | 4.51 | 11.61 | 3.91 |
| 115 | | | 1702 | Rejang Lebong | 2.75 | 1.88 | 3.87 | 0.38 | 0.12 | 0.91 | 15.60 | 2.40 | 55.50 | 127.00 | 13.85 | 7.08 | 18.10 | 5.63 |
| 116 | | | 1703 | Bengkulu Utara | 2.17 | 1.41 | 3.19 | 0.71 | 0.30 | 1.41 | 18.90 | 2.40 | 69.50 | 150.40 | 17.78 | 6.46 | 54.52 | 28.67 |
| 117 | | | 1704 | Kaur | 1.88 | 1.14 | 2.92 | 0.53 | 0.19 | 1.17 | 27.80 | 4.17 | 84.30 | 147.80 | 9.89 | 6.46 | 47.08 | 17.52 |
| 118 | | | 1705 | Seluma | 1.98 | 1.23 | 3.01 | 0.34 | 0.09 | 0.88 | 11.40 | 3.57 | 33.50 | 89.20 | 16.48 | 6.47 | 41.10 | 41.61 |
| 119 | | | 1706 | Mukomuko | 5.80 | 4.44 | 7.42 | 1.05 | 0.55 | 1.80 | 29.50 | 3.07 | 66.90 | 222.90 | 12.19 | 5.49 | 51.76 | 22.33 |
| 120 | | | 1707 | Lebong | 2.91 | 1.96 | 4.15 | 1.00 | 0.47 | 1.86 | 18.90 | 3.97 | 27.40 | 128.60 | 9.52 | 6.50 | 41.56 | 33.31 |
| 121 | | | 1708 | Kepahiang | 3.46 | 2.38 | 4.86 | 0.74 | 0.29 | 1.52 | 13.50 | 3.16 | 85.70 | 129.30 | 14.00 | 6.85 | 27.68 | 16.09 |
| 122 | | | 1709 | Bengkulu Tengah | 3.05 | 2.04 | 4.37 | 0.57 | 0.18 | 1.34 | 29.60 | 5.73 | 17.20 | 125.00 | 11.61 | 4.03 | 42.59 | 17.12 |
| 123 | | | 1771 | Bengkulu | 2.60 | 1.74 | 3.71 | 1.82 | 1.13 | 2.76 | 40.40 | 1.82 | 315.10 | 313.80 | 12.06 | 3.92 | 24.86 | 7.27 |
| 124 | 18 | Lampung | 1801 | Lampung Barat | 2.15 | 1.47 | 3.02 | 0.50 | 0.20 | 1.00 | 12.10 | 1.43 | 170.60 | 66.50 | 20.04 | 5.09 | 96.42 | 56.61 |
| 125 | | | 1802 | Tanggamus | 3.34 | 2.45 | 4.45 | 0.70 | 0.31 | 1.31 | 6.80 | 1.23 | 61.20 | 41.40 | 36.11 | 6.57 | 75.24 | 30.91 |
| 126 | | | 1803 | Lampung Selatan | 3.93 | 3.04 | 5.00 | 1.04 | 0.61 | 1.64 | 7.40 | 0.82 | 50.20 | 49.30 | 27.85 | 7.21 | 61.08 | 16.33 |
| 127 | | | 1804 | Lampung Timur | 6.44 | 5.31 | 7.74 | 0.84 | 0.47 | 1.39 | 7.00 | 0.99 | 16.00 | 36.30 | 14.59 | 4.67 | 59.86 | 54.02 |
| 128 | | | 1805 | Lampung Tengah | 3.47 | 2.66 | 4.46 | 0.76 | 0.41 | 1.30 | 10.60 | 0.90 | 44.00 | 67.60 | 21.32 | 4.55 | 88.02 | 30.27 |
| 129 | | | 1806 | Lampung Utara | 7.54 | 6.16 | 9.12 | 1.14 | 0.64 | 1.86 | 11.10 | 1.27 | 65.60 | 189.10 | 22.20 | 3.84 | 31.90 | 9.34 |
| 130 | | | 1807 | Way Kanan | 2.94 | 2.12 | 3.97 | 0.76 | 0.39 | 1.33 | 14.80 | 1.34 | 53.60 | 92.60 | 18.83 | 4.21 | 58.87 | 13.16 |
| 131 | | | 1808 | Tulang Bawang | 1.94 | 1.24 | 2.89 | 0.61 | 0.27 | 1.17 | 11.50 | 1.30 | 33.80 | 82.30 | 21.92 | 4.39 | 52.89 | 29.43 |
| 132 | | | 1809 | Pesawaran | 4.93 | 3.87 | 6.19 | 0.77 | 0.38 | 1.38 | 7.40 | 0.86 | 0.00 | 26.60 | 20.10 | 3.92 | 76.16 | 18.59 |
| 133 | | | 1810 | Pringsewu | 0.60 | 0.27 | 1.14 | 0.14 | 0.02 | 0.50 | 12.80 | 0.86 | 22.20 | 110.20 | 19.07 | 4.18 | 56.88 | 7.62 |
| 134 | | | 1811 | Mesuji | 1.29 | 0.71 | 2.17 | 0.48 | 0.15 | 1.12 | 10.70 | 1.83 | 0.00 | 86.60 | 18.24 | 5.36 | 103.04 | 58.81 |
| 135 | | | 1812 | Tulang Bawang Barat | 1.89 | 1.17 | 2.88 | 0.70 | 0.32 | 1.34 | 5.30 | 1.03 | 0.00 | 46.10 | 17.22 | 4.16 | 51.88 | 13.97 |

| | | | | | | | | | | | | | | | | | | |
|---|---|---|---|---|---|---|---|---|---|---|---|---|---|---|---|---|---|---|
| 136 | | | 1871 | Bandar Lampung | 4.10 | 3.20 | 5.17 | 1.76 | 1.20 | 2.49 | 29.20 | 0.97 | 222.60 | 156.00 | 21.57 | 5.67 | 31.73 | 7.43 |
| 137 | | | 1872 | Metro | 3.97 | 2.94 | 5.25 | 2.08 | 1.35 | 3.05 | 57.70 | 2.16 | 364.10 | 300.50 | 12.77 | 4.51 | 19.87 | 6.75 |
| 138 | 19 | Bangka Belitung | 1901 | Bangka | 8.90 | 7.25 | 10.83 | 2.48 | 1.65 | 3.59 | 28.10 | 1.15 | 143.50 | 168.40 | 14.53 | 5.58 | 37.95 | 36.00 |
| 139 | | | 1902 | Belitung | 6.50 | 5.12 | 8.13 | 2.55 | 1.70 | 3.68 | 23.30 | 1.53 | 164.20 | 160.80 | 16.38 | 5.95 | 30.63 | 12.52 |
| 140 | | | 1903 | Bangka Barat | 6.66 | 5.12 | 8.50 | 0.89 | 0.40 | 1.70 | 15.20 | 1.21 | 69.30 | 162.90 | 17.57 | 6.10 | 34.89 | 10.83 |
| 141 | | | 1904 | Bangka Tengah | 7.77 | 6.11 | 9.74 | 1.73 | 1.02 | 2.72 | 19.20 | 1.32 | 33.50 | 135.20 | 12.88 | 6.41 | 34.70 | 16.87 |
| 142 | | | 1905 | Bangka Selatan | 10.28 | 8.36 | 12.51 | 2.62 | 1.70 | 3.86 | 14.90 | 1.39 | 36.50 | 131.50 | 10.47 | 4.66 | 52.34 | 83.08 |
| 143 | | | 1906 | Belitung Timur | 3.94 | 2.76 | 5.44 | 2.15 | 1.30 | 3.34 | 25.00 | 1.50 | 61.60 | 268.80 | 18.19 | 8.06 | 44.88 | 20.75 |
| 144 | | | 1971 | Pangkal Pinang | 12.25 | 10.26 | 14.51 | 3.21 | 2.22 | 4.50 | 42.10 | 1.37 | 196.20 | 303.20 | 7.91 | 2.38 | 14.63 | 5.07 |
| 145 | 21 | Kepulauan Riau | 2101 | Karimun | 10.63 | 8.83 | 12.69 | 1.26 | 0.70 | 2.09 | 32.70 | 1.05 | 92.10 | 200.20 | 12.59 | 6.63 | 71.15 | 68.11 |
| 146 | | | 2102 | Bintan | 6.31 | 4.84 | 8.07 | 2.62 | 1.73 | 3.81 | 52.40 | 2.44 | 67.90 | 273.00 | 18.57 | 7.69 | 50.68 | 29.46 |
| 147 | | | 2103 | Natuna | 3.55 | 2.37 | 5.12 | 2.80 | 1.70 | 4.31 | 73.10 | 4.67 | 183.20 | 541.30 | 21.24 | 5.93 | 57.36 | 24.33 |
| 148 | | | 2104 | Lingga | 7.18 | 5.56 | 9.13 | 1.13 | 0.56 | 2.03 | 35.50 | 2.01 | 96.80 | 406.30 | 16.76 | 6.43 | 25.49 | 13.11 |
| 149 | | | 2105 | Kepulauan Anambas | 14.58 | 11.89 | 17.68 | 2.68 | 1.59 | 4.19 | 64.10 | 4.64 | 30.90 | 538.20 | 21.67 | 19.19 | 48.38 | 50.29 |
| 150 | | | 2171 | Batam | 6.93 | 5.31 | 8.84 | 3.64 | 2.46 | 5.13 | 13.70 | 0.65 | 124.90 | 89.00 | 18.12 | 7.90 | 38.56 | 20.36 |
| 151 | | | 2172 | Tanjung Pinang | 8.41 | 6.72 | 10.37 | 1.93 | 1.19 | 2.97 | 31.30 | 0.79 | 215.70 | 348.50 | 10.78 | 4.70 | 17.34 | 6.46 |
| 152 | 31 | DKI Jakarta | 3101 | Kepulauan Seribu | 23.00 | 18.65 | 28.00 | 0.35 | 0.04 | 1.27 | 107.90 | 10.79 | 67.40 | 602.50 | 18.14 | 1.53 | 120.27 | 125.76 |
| 153 | | | 3171 | Jakarta Selatan | 11.86 | 10.12 | 13.81 | 3.27 | 2.38 | 4.39 | 24.20 | 1.08 | 204.10 | 160.80 | 13.19 | 2.66 | 28.66 | 7.27 |
| 154 | | | 3172 | Jakarta Timur | 13.09 | 11.49 | 14.84 | 3.70 | 2.89 | 4.67 | 23.50 | 0.93 | 197.60 | 158.00 | 11.08 | 1.90 | 23.27 | 5.48 |
| 155 | | | 3173 | Jakarta Pusat | 6.24 | 5.04 | 7.64 | 1.75 | 1.14 | 2.56 | 60.70 | 1.32 | 651.20 | 587.50 | 11.39 | 3.24 | 23.45 | 9.50 |
| 156 | | | 3174 | Jakarta Barat | 7.00 | 5.91 | 8.23 | 1.78 | 1.27 | 2.41 | 15.70 | 0.93 | 150.10 | 121.60 | 13.20 | 4.90 | 28.78 | 12.47 |
| 157 | | | 3175 | Jakarta Utara | 6.81 | 5.62 | 8.17 | 1.59 | 1.04 | 2.31 | 26.00 | 0.85 | 162.80 | 154.80 | 14.01 | 2.24 | 36.62 | 7.99 |
| 158 | 32 | Jawa Barat | 3201 | Bogor | 7.04 | 10.96 | 13.86 | 2.57 | 1.97 | 3.29 | 8.70 | 0.59 | 69.40 | 36.00 | 16.61 | 3.79 | 43.03 | 11.32 |
| 159 | | | 3202 | Sukabumi | 6.86 | 5.98 | 8.22 | 1.78 | 1.27 | 2.42 | 6.20 | 0.69 | 266.20 | 29.00 | 20.32 | 5.96 | 41.36 | 9.28 |
| 160 | | | 3203 | Cianjur | 13.28 | 5.79 | 8.08 | 1.11 | 0.71 | 1.65 | 4.80 | 0.58 | 20.40 | 34.60 | 34.33 | 5.11 | 136.57 | 18.14 |
| 161 | | | 3204 | Bandung | 5.03 | 11.83 | 14.86 | 3.27 | 2.58 | 4.10 | 4.50 | 0.54 | 31.00 | 31.50 | 17.79 | 2.70 | 48.12 | 12.08 |
| 162 | | | 3205 | Garut | 8.35 | 4.18 | 6.01 | 0.24 | 0.09 | 0.53 | 3.90 | 0.75 | 32.60 | 48.20 | 27.00 | 5.23 | 73.82 | 19.90 |
| 163 | | | 3206 | Tasikmalaya | 6.94 | 7.06 | 9.79 | 1.02 | 0.60 | 1.60 | 4.30 | 0.67 | 40.50 | 26.70 | 20.99 | 4.33 | 89.02 | 15.28 |
| 164 | | | 3207 | Ciamis | 10.14 | 5.85 | 8.16 | 1.27 | 0.85 | 1.82 | 6.10 | 0.95 | 41.00 | 43.60 | 21.03 | 4.05 | 58.00 | 21.31 |
| 165 | | | 3208 | Kuningan | 6.99 | 8.76 | 11.67 | 2.09 | 1.48 | 2.85 | 10.20 | 1.00 | 82.90 | 56.00 | 18.00 | 3.17 | 58.05 | 10.59 |
| 166 | | | 3209 | Cirebon | 11.95 | 5.96 | 8.13 | 1.39 | 0.95 | 1.95 | 10.00 | 0.77 | 69.90 | 78.00 | 17.72 | 3.01 | 46.72 | 8.69 |
| 167 | | | 3210 | Majalengka | 7.78 | 10.34 | 13.74 | 1.71 | 1.14 | 2.45 | 6.90 | 0.77 | 41.90 | 59.70 | 17.01 | 2.88 | 52.52 | 10.26 |
| 168 | | | 3211 | Sumedang | 7.63 | 6.56 | 9.15 | 2.20 | 1.57 | 2.99 | 5.30 | 0.82 | 32.00 | 61.30 | 24.98 | 3.79 | 58.80 | 13.37 |
| 169 | | | 3212 | Indramayu | 8.83 | 6.44 | 8.97 | 1.57 | 1.03 | 2.26 | 6.30 | 0.82 | 27.60 | 53.70 | 15.62 | 3.28 | 37.93 | 9.25 |
| 170 | | | 3213 | Subang | 10.05 | 7.60 | 10.22 | 2.11 | 1.52 | 2.85 | 6.50 | 0.76 | 43.90 | 64.50 | 19.14 | 4.70 | 54.35 | 23.07 |
| 171 | | | 3214 | Purwakarta | 3.91 | 8.55 | 11.74 | 3.24 | 2.42 | 4.25 | 11.30 | 0.65 | 100.00 | 104.70 | 20.54 | 3.07 | 42.68 | 6.96 |
| 172 | | | 3215 | Karawang | 5.24 | 3.12 | 4.83 | 0.99 | 0.62 | 1.48 | 9.40 | 0.66 | 84.90 | 90.30 | 15.60 | 4.82 | 38.36 | 9.53 |
| 173 | | | 3216 | Bekasi | 9.65 | 4.31 | 6.31 | 2.00 | 1.44 | 2.71 | 10.00 | 0.41 | 99.40 | 58.40 | 17.65 | 3.38 | 41.65 | 11.84 |
| 174 | | | 3217 | Bandung Barat | 9.65 | 8.30 | 11.15 | 1.65 | 1.13 | 2.33 | 5.80 | 0.57 | 31.80 | 29.80 | 21.15 | 4.10 | 42.30 | 11.13 |
| 175 | | | 3271 | Kota Bogor | 21.35 | 19.07 | 23.82 | 2.28 | 1.58 | 3.19 | 24.00 | 0.70 | 192.10 | 120.40 | 13.98 | 1.20 | 25.38 | 3.56 |
| 176 | | | 3272 | Kota Sukabumi | 23.71 | 21.10 | 26.56 | 5.12 | 3.95 | 6.52 | 25.20 | 1.40 | 266.20 | 198.00 | 12.09 | 1.44 | 24.80 | 3.33 |
| 177 | | | 3273 | Kota Bandung | 5.11 | 4.27 | 6.07 | 0.87 | 0.54 | 1.33 | 22.30 | 0.85 | 202.20 | 199.20 | 15.97 | 2.99 | 34.30 | 6.39 |
| 178 | | | 3274 | Kota Cirebon | 8.53 | 7.00 | 10.29 | 1.71 | 1.03 | 2.65 | 43.90 | 2.07 | 348.90 | 289.90 | 12.62 | 3.00 | 16.93 | 4.83 |
| 179 | | | 3275 | Kota Bekasi | 13.01 | 11.54 | 14.61 | 3.72 | 2.97 | 4.60 | 13.10 | 0.37 | 111.00 | 59.60 | 15.99 | 3.42 | 25.41 | 7.45 |
| 180 | | | 3276 | Depok | 12.47 | 10.81 | 14.31 | 3.12 | 2.31 | 4.12 | 11.70 | 0.51 | 50.20 | 44.80 | 14.20 | 2.96 | 26.07 | 5.42 |
| 181 | | | 3277 | Cimahi | 14.27 | 12.34 | 16.42 | 4.42 | 3.38 | 5.69 | 25.10 | 0.67 | 198.10 | 163.40 | 11.49 | 1.72 | 23.46 | 3.57 |
| 182 | | | 3278 | Kota Tasikmalaya | 17.32 | 15.30 | 19.54 | 1.59 | 1.02 | 2.35 | 16.50 | 0.88 | 69.70 | 135.80 | 14.64 | 1.96 | 37.66 | 3.55 |
| 183 | | | 3279 | Banjar | 5.36 | 4.10 | 6.86 | 1.70 | 1.02 | 2.66 | 21.80 | 1.59 | 46.20 | 208.60 | 13.08 | 2.29 | 20.74 | 3.36 |

| | | | | | | | | | | | | | | | | | | |
|---|---|---|---|---|---|---|---|---|---|---|---|---|---|---|---|---|---|---|
| 184 | 33 | Jawa Tengah | 3301 | Cilacap | 7.39 | 6.23 | 8.71 | 2.09 | 1.47 | 2.86 | 10.20 | 0.69 | 52.70 | 81.80 | 24.10 | 3.31 | 59.33 | 16.11 |
| 185 | | | 3302 | Banyumas | 8.13 | 6.92 | 9.48 | 4.27 | 3.38 | 5.31 | 14.70 | 0.74 | 172.70 | 123.50 | 14.78 | 2.89 | 32.13 | 5.45 |
| 186 | | | 3303 | Purbalingga | 12.86 | 11.15 | 14.75 | 2.83 | 2.05 | 3.79 | 10.90 | 0.77 | 71.10 | 73.80 | 17.08 | 3.29 | 41.27 | 10.02 |
| 187 | | | 3304 | Banjarnegara | 8.97 | 7.45 | 10.69 | 3.04 | 2.20 | 4.09 | 9.00 | 1.19 | 54.00 | 50.90 | 17.49 | 4.51 | 49.67 | 10.64 |
| 188 | | | 3305 | Kebumen | 8.16 | 6.89 | 9.59 | 3.02 | 2.24 | 3.96 | 10.90 | 0.89 | 100.40 | 86.40 | 15.32 | 2.17 | 37.78 | 5.15 |
| 189 | | | 3306 | Purworejo | 5.19 | 4.07 | 6.50 | 2.66 | 1.85 | 3.68 | 24.10 | 1.15 | 106.70 | 160.40 | 21.43 | 3.52 | 47.35 | 7.22 |
| 190 | | | 3307 | Wonosobo | 6.25 | 5.12 | 7.55 | 1.63 | 1.09 | 2.34 | 9.30 | 0.94 | 62.30 | 58.40 | 17.75 | 2.57 | 38.82 | 5.70 |
| 191 | | | 3308 | Magelang | 11.64 | 10.09 | 13.36 | 2.54 | 1.84 | 3.40 | 11.90 | 0.73 | 21.10 | 36.40 | 15.09 | 1.91 | 30.67 | 3.91 |
| 192 | | | 3309 | Boyolali | 11.31 | 9.72 | 13.08 | 5.16 | 4.09 | 6.41 | 14.80 | 0.92 | 114.90 | 82.20 | 21.48 | 2.77 | 33.88 | 7.08 |
| 193 | | | 3310 | Klaten | 10.12 | 8.61 | 11.81 | 3.16 | 2.32 | 4.19 | 12.60 | 0.89 | 106.10 | 108.80 | 14.57 | 3.34 | 26.57 | 7.19 |
| 194 | | | 3311 | Sukoharjo | 2.46 | 1.76 | 3.35 | 1.43 | 0.90 | 2.15 | 15.30 | 0.43 | 80.90 | 67.10 | 14.10 | 3.45 | 28.76 | 7.51 |
| 195 | | | 3312 | Wonogiri | 10.45 | 8.91 | 12.16 | 2.95 | 2.16 | 3.91 | 14.10 | 1.08 | 79.20 | 75.00 | 14.82 | 2.22 | 36.72 | 8.53 |
| 196 | | | 3313 | Karanganyar | 9.23 | 7.88 | 10.73 | 4.07 | 3.19 | 5.11 | 15.10 | 0.77 | 84.70 | 74.60 | 12.95 | 4.62 | 40.05 | 16.09 |
| 197 | | | 3314 | Sragen | 6.55 | 5.37 | 7.90 | 2.76 | 2.01 | 3.69 | 17.70 | 0.86 | 119.10 | 83.30 | 15.18 | 3.58 | 32.75 | 8.08 |
| 198 | | | 3315 | Grobogan | 9.60 | 8.24 | 11.10 | 2.58 | 1.90 | 3.42 | 10.70 | 0.68 | 78.10 | 80.20 | 18.33 | 3.23 | 52.86 | 6.90 |
| 199 | | | 3316 | Blora | 3.93 | 3.07 | 4.95 | 1.24 | 0.79 | 1.85 | 13.10 | 0.93 | 72.70 | 68.60 | 20.26 | 5.17 | 50.03 | 26.71 |
| 200 | | | 3317 | Rembang | 4.99 | 3.94 | 6.24 | 0.75 | 0.38 | 1.32 | 8.90 | 0.80 | 55.10 | 65.70 | 16.89 | 2.47 | 43.81 | 6.79 |
| 201 | | | 3318 | Pati | 6.55 | 5.30 | 8.00 | 2.73 | 1.94 | 3.73 | 13.00 | 0.72 | 83.50 | 91.20 | 22.12 | 3.87 | 43.60 | 8.58 |
| 202 | | | 3319 | Kudus | 12.03 | 10.50 | 13.73 | 2.09 | 1.49 | 2.85 | 19.90 | 0.72 | 170.40 | 144.00 | 11.83 | 1.83 | 38.68 | 3.65 |
| 203 | | | 3320 | Jepara | 10.70 | 9.26 | 12.29 | 3.30 | 2.52 | 4.25 | 13.30 | 0.57 | 79.00 | 67.10 | 13.98 | 3.22 | 44.42 | 10.35 |
| 204 | | | 3321 | Demak | 9.29 | 7.96 | 10.78 | 3.01 | 2.28 | 3.90 | 11.00 | 0.76 | 48.30 | 38.50 | 14.60 | 2.58 | 40.68 | 6.45 |
| 205 | | | 3322 | Semarang | 9.03 | 7.67 | 10.57 | 3.59 | 2.76 | 4.57 | 14.50 | 0.83 | 74.20 | 76.80 | 13.01 | 2.12 | 33.94 | 4.73 |
| 206 | | | 3323 | Temanggung | 7.27 | 6.05 | 8.66 | 2.15 | 1.51 | 2.97 | 11.70 | 1.00 | 78.60 | 90.20 | 17.51 | 2.62 | 35.29 | 11.17 |
| 207 | | | 3324 | Kendal | 6.20 | 5.13 | 7.43 | 0.97 | 0.58 | 1.52 | 12.60 | 0.99 | 41.80 | 84.10 | 16.81 | 2.54 | 45.09 | 7.67 |
| 208 | | | 3325 | Batang | 10.31 | 8.91 | 11.87 | 2.09 | 1.48 | 2.86 | 9.20 | 0.88 | 58.80 | 76.20 | 13.67 | 1.58 | 51.52 | 4.61 |
| 209 | | | 3326 | Pekalongan | 10.00 | 8.51 | 11.67 | 2.05 | 1.39 | 2.91 | 11.30 | 0.92 | 93.80 | 72.60 | 14.83 | 1.79 | 45.19 | 7.10 |
| 210 | | | 3327 | Pemalang | 7.28 | 6.05 | 8.68 | 2.80 | 2.04 | 3.76 | 9.90 | 0.52 | 63.30 | 57.70 | 19.28 | 4.11 | 44.27 | 34.43 |
| 211 | | | 3328 | Tegal | 16.48 | 14.61 | 18.51 | 2.35 | 1.66 | 3.22 | 6.40 | 0.62 | 57.80 | 41.30 | 20.24 | 2.73 | 50.67 | 10.71 |
| 212 | | | 3329 | Brebes | 8.81 | 7.49 | 10.28 | 1.37 | 0.88 | 2.03 | 8.00 | 0.65 | 46.40 | 43.80 | 21.03 | 4.23 | 46.00 | 13.95 |
| 213 | | | 3371 | Kota Magelang | 15.91 | 13.54 | 18.55 | 5.36 | 4.01 | 7.00 | 83.60 | 1.25 | ###### | 758.70 | 8.90 | 1.04 | 13.33 | 1.93 |
| 214 | | | 3372 | Surakarta | 10.51 | 8.92 | 12.29 | 3.58 | 2.67 | 4.69 | 63.30 | 1.01 | 625.80 | 535.00 | 9.50 | 1.58 | 16.22 | 3.01 |
| 215 | | | 3373 | Salatiga | 17.18 | 14.83 | 19.78 | 4.21 | 3.08 | 5.59 | 74.80 | 1.04 | 441.40 | 305.10 | 9.93 | 1.36 | 14.28 | 2.19 |
| 216 | | | 3374 | Kota Semarang | 10.26 | 8.96 | 11.70 | 3.95 | 3.15 | 4.88 | 73.50 | 0.70 | 305.40 | 226.00 | 12.49 | 2.74 | 31.71 | 6.28 |
| 217 | | | 3375 | Kota Pekalongan | 9.99 | 8.38 | 11.82 | 2.43 | 1.66 | 3.43 | 25.60 | 1.47 | 178.00 | 107.10 | 11.35 | 2.69 | 17.44 | 4.29 |
| 218 | | | 3376 | Kota Tegal | 12.34 | 10.45 | 14.48 | 1.89 | 1.19 | 2.83 | 33.40 | 0.99 | 362.40 | 325.70 | 12.83 | 3.11 | 22.07 | 6.13 |
| 219 | 34 | DI Yogyakarta | 3401 | Kulonprogo | 17.88 | 15.80 | 20.14 | 4.52 | 3.48 | 5.76 | 23.30 | 1.56 | 126.90 | 109.10 | 14.08 | 1.77 | 25.71 | 4.21 |
| 220 | | | 3402 | Bantul | 13.64 | 11.92 | 15.52 | 4.10 | 3.19 | 5.19 | 29.80 | 0.85 | 88.20 | 103.40 | 13.40 | 2.16 | 21.67 | 3.93 |
| 221 | | | 3403 | Gunung Kidul | 13.76 | 11.93 | 15.77 | 2.88 | 2.11 | 3.81 | 28.50 | 1.28 | 43.70 | 97.00 | 14.26 | 2.50 | 32.57 | 8.82 |
| 222 | | | 3404 | Sleman | 12.89 | 11.24 | 14.70 | 3.36 | 2.54 | 4.36 | 33.50 | 0.66 | 571.60 | 204.30 | 14.70 | 3.05 | 29.54 | 5.51 |
| 223 | | | 3471 | Yogyakarta | 13.49 | 11.65 | 15.53 | 5.17 | 4.05 | 6.50 | 108.70 | 1.34 | 568.80 | 573.00 | 8.64 | 2.05 | 14.59 | 4.42 |
| 224 | 35 | Jawa Timur | 3501 | Pacitan | 6.27 | 5.07 | 7.65 | 2.59 | 1.79 | 3.60 | 12.00 | 1.29 | 35.10 | 85.20 | 19.23 | 3.02 | 71.60 | 20.85 |
| 225 | | | 3502 | Ponorogo | 6.40 | 5.27 | 7.69 | 1.75 | 1.22 | 2.42 | 9.70 | 1.06 | 94.50 | 103.50 | 20.07 | 3.72 | 42.57 | 10.86 |
| 226 | | | 3503 | Trenggalek | 6.53 | 5.38 | 7.84 | 1.42 | 0.93 | 2.05 | 12.80 | 0.95 | 66.80 | 84.10 | 15.18 | 3.03 | 47.00 | 8.65 |
| 227 | | | 3504 | Tulungagung | 6.43 | 5.32 | 7.69 | 1.71 | 1.16 | 2.40 | 9.80 | 0.91 | 90.20 | 93.30 | 14.54 | 2.35 | 35.37 | 8.16 |
| 228 | | | 3505 | Blitar | 7.70 | 6.50 | 9.06 | 2.56 | 1.88 | 3.39 | 6.30 | 0.63 | 42.10 | 41.60 | 16.49 | 2.46 | 33.82 | 6.49 |
| 229 | | | 3506 | Kediri | 9.30 | 8.05 | 10.69 | 2.31 | 1.71 | 3.04 | 8.50 | 0.72 | 53.40 | 69.30 | 15.00 | 2.71 | 37.22 | 7.13 |
| 230 | | | 3507 | Malang | 6.77 | 5.77 | 7.89 | 2.37 | 1.78 | 3.08 | 10.00 | 0.47 | 90.00 | 44.00 | 16.25 | 2.79 | 38.47 | 8.86 |
| 231 | | | 3508 | Lumajang | 7.81 | 6.60 | 9.17 | 2.03 | 1.43 | 2.79 | 10.00 | 0.72 | 50.40 | 68.30 | 18.21 | 3.23 | 39.96 | 9.07 |

| | | | | | | | | | | | | | | | | | | |
|---|---|---|---|---|---|---|---|---|---|---|---|---|---|---|---|---|---|---|
| 232 | | | 3509 | Jember | 7.17 | 6.18 | 8.28 | 1.49 | 1.07 | 2.03 | 8.60 | 0.61 | 63.30 | 55.80 | 18.03 | 3.87 | 47.31 | 9.82 |
| 233 | | | 3510 | Banyuwangi | 8.62 | 7.46 | 9.90 | 2.65 | 2.01 | 3.44 | 7.10 | 0.84 | 71.00 | 52.40 | 15.16 | 3.02 | 40.57 | 8.10 |
| 234 | | | 3511 | Bondowoso | 6.39 | 5.20 | 7.75 | 0.93 | 0.53 | 1.52 | 9.10 | 0.99 | 37.60 | 131.80 | 21.07 | 5.42 | 30.38 | 6.86 |
| 235 | | | 3512 | Situbondo | 7.65 | 6.39 | 9.09 | 2.93 | 2.16 | 3.87 | 8.00 | 0.77 | 60.10 | 75.40 | 16.54 | 2.52 | 48.83 | 6.59 |
| 236 | | | 3513 | Probolinggo | 7.02 | 5.89 | 8.29 | 1.75 | 1.21 | 2.45 | 6.10 | 0.88 | 61.50 | 51.20 | 17.27 | 3.18 | 36.54 | 6.78 |
| 237 | | | 3514 | Pasuruan | 6.44 | 5.43 | 7.59 | 1.46 | 1.00 | 2.06 | 6.00 | 0.64 | 45.30 | 27.10 | 19.63 | 3.33 | 37.30 | 7.36 |
| 238 | | | 3515 | Sidoarjo | 11.84 | 10.53 | 13.27 | 4.09 | 3.34 | 4.96 | 13.80 | 0.39 | 114.20 | 78.60 | 13.07 | 1.70 | 31.01 | 4.90 |
| 239 | | | 3516 | Mojokerto | 6.04 | 4.95 | 7.28 | 2.01 | 1.40 | 2.78 | 13.90 | 0.77 | 112.50 | 107.80 | 16.36 | 3.94 | 36.93 | 7.74 |
| 240 | | | 3517 | Jombang | 9.59 | 8.26 | 11.07 | 2.32 | 1.69 | 3.11 | 13.30 | 0.83 | 89.50 | 87.20 | 12.51 | 2.64 | 32.52 | 5.86 |
| 241 | | | 3518 | Nganjuk | 5.45 | 4.47 | 6.58 | 1.20 | 0.76 | 1.80 | 11.70 | 0.57 | 57.60 | 61.30 | 20.91 | 3.77 | 38.56 | 8.26 |
| 242 | | | 3519 | Madiun | 7.16 | 5.86 | 8.65 | 2.29 | 1.59 | 3.17 | 23.60 | 1.15 | 66.20 | 172.40 | 17.24 | 3.05 | 29.96 | 6.77 |
| 243 | | | 3520 | Magetan | 9.48 | 8.08 | 11.04 | 2.24 | 1.59 | 3.05 | 8.50 | 1.03 | 77.10 | 60.50 | 13.51 | 1.97 | 35.92 | 9.18 |
| 244 | | | 3521 | Ngawi | 8.90 | 7.54 | 10.42 | 3.14 | 2.30 | 4.18 | 10.10 | 0.86 | 46.50 | 96.10 | 13.97 | 2.15 | 37.73 | 16.22 |
| 245 | | | 3522 | Bojonegoro | 6.20 | 5.19 | 7.33 | 1.97 | 1.43 | 2.65 | 8.50 | 0.87 | 71.10 | 57.80 | 17.25 | 3.19 | 37.92 | 5.86 |
| 246 | | | 3523 | Tuban | 5.61 | 4.66 | 6.69 | 1.47 | 1.01 | 2.07 | 6.30 | 0.86 | 71.80 | 40.00 | 17.57 | 1.92 | 47.79 | 7.74 |
| 247 | | | 3524 | Lamongan | 8.77 | 7.55 | 10.13 | 1.99 | 1.43 | 2.70 | 8.20 | 0.82 | 46.00 | 69.90 | 21.87 | 5.43 | 46.63 | 11.49 |
| 248 | | | 3525 | Gresik | 13.39 | 11.82 | 15.10 | 3.61 | 2.83 | 4.56 | 12.60 | 0.79 | 70.10 | 60.80 | 16.75 | 3.07 | 36.92 | 7.21 |
| 249 | | | 3526 | Bangkalan | 2.80 | 2.03 | 3.74 | 0.94 | 0.52 | 1.56 | 7.30 | 0.71 | 37.50 | 62.50 | 19.04 | 3.69 | 44.20 | 8.89 |
| 250 | | | 3527 | Sampang | 7.54 | 6.24 | 9.03 | 2.37 | 1.68 | 3.26 | 5.00 | 0.70 | 19.30 | 46.80 | 18.07 | 2.52 | 64.08 | 9.41 |
| 251 | | | 3528 | Pamekasan | 5.11 | 4.08 | 6.32 | 0.85 | 0.46 | 1.43 | 7.50 | 0.73 | 36.30 | 69.50 | 24.10 | 3.87 | 26.51 | 7.28 |
| 252 | | | 3529 | Sumenep | 8.23 | 6.95 | 9.67 | 1.37 | 0.89 | 2.02 | 6.40 | 0.84 | 23.30 | 65.40 | 21.00 | 2.60 | 119.90 | 15.84 |
| 253 | | | 3571 | Kota Kediri | 8.60 | 7.16 | 10.25 | 1.90 | 1.27 | 2.74 | 35.10 | 0.98 | 495.90 | 348.50 | 9.49 | 3.19 | 19.66 | 5.30 |
| 254 | | | 3572 | Kota Blitar | 13.04 | 10.92 | 15.43 | 3.55 | 2.50 | 4.89 | 45.70 | 0.66 | 430.40 | 261.70 | 8.32 | 2.26 | 12.62 | 3.08 |
| 255 | | | 3573 | Kota Malang | 8.86 | 7.57 | 10.31 | 1.79 | 1.25 | 2.49 | 23.20 | 0.53 | 308.20 | 241.00 | 10.31 | 1.56 | 17.77 | 2.80 |
| 256 | | | 3574 | Kota Probolinggo | 13.25 | 11.28 | 15.46 | 1.96 | 1.26 | 2.91 | 17.90 | 0.85 | 142.50 | 145.60 | 13.12 | 2.16 | 22.48 | 3.64 |
| 257 | | | 3575 | Kota Pasuruan | 12.68 | 10.68 | 14.95 | 3.97 | 2.89 | 5.30 | 29.80 | 1.21 | 141.50 | 230.80 | 11.40 | 1.44 | 16.68 | 2.95 |
| 258 | | | 3576 | Kota Mojokerto | 15.77 | 13.45 | 18.38 | 4.77 | 3.52 | 6.31 | 28.30 | 1.21 | 577.80 | 165.10 | 11.17 | 1.74 | 16.33 | 2.84 |
| 259 | | | 3577 | Kota Madiun | 9.52 | 7.89 | 11.36 | 3.41 | 2.46 | 4.61 | 20.50 | 1.02 | 527.40 | 46.70 | 9.23 | 2.22 | 15.66 | 4.02 |
| 260 | | | 3578 | Surabaya | 15.39 | 13.85 | 17.05 | 4.29 | 3.49 | 5.22 | 25.40 | 0.65 | 293.70 | 188.50 | 8.92 | 1.64 | 20.96 | 4.79 |
| 261 | | | 3579 | Batu | 10.74 | 8.98 | 12.73 | 2.32 | 1.55 | 3.32 | 32.70 | 0.77 | 149.60 | 138.60 | 10.80 | 1.50 | 15.45 | 2.00 |
| 262 | 36 | Banten | 3601 | Pandeglang | 7.60 | 6.37 | 9.00 | 0.71 | 0.37 | 1.21 | 4.70 | 0.84 | 17.40 | 62.70 | 23.52 | 4.53 | 73.92 | 13.45 |
| 263 | | | 3602 | Lebak | 6.16 | 5.14 | 7.32 | 1.26 | 0.83 | 1.84 | 8.20 | 0.90 | 47.50 | 57.30 | 14.40 | 4.65 | 54.65 | 11.85 |
| 264 | | | 3603 | Tangerang | 8.41 | 7.25 | 9.69 | 2.60 | 1.99 | 3.34 | 8.50 | 0.41 | 62.90 | 38.50 | 24.59 | 4.10 | 53.39 | 17.63 |
| 265 | | | 3604 | Serang | 6.25 | 5.15 | 7.50 | 0.88 | 0.51 | 1.41 | 9.50 | 0.60 | 42.50 | 94.00 | 21.93 | 5.99 | 59.03 | 17.37 |
| 266 | | | 3671 | Kota Tangerang | 10.08 | 8.63 | 11.69 | 3.15 | 2.36 | 4.10 | 14.40 | 0.48 | 150.50 | 74.70 | 11.19 | 1.93 | 25.65 | 4.30 |
| 267 | | | 3672 | Cilegon | 10.17 | 8.45 | 12.13 | 2.77 | 1.94 | 3.83 | 23.30 | 0.58 | 130.70 | 101.50 | 14.47 | 4.95 | 29.61 | 10.20 |
| 268 | | | 3673 | Kota Serang | 8.25 | 6.71 | 10.02 | 2.25 | 1.42 | 3.35 | 16.30 | 0.75 | 53.20 | 81.20 | 12.47 | 3.87 | 29.14 | 6.65 |
| 269 | | | 3674 | Tangerang Selatan | 5.20 | 4.24 | 6.32 | 1.65 | 1.13 | 2.32 | 17.00 | 0.52 | 106.00 | 82.90 | 15.27 | 5.17 | 26.34 | 8.70 |
| 270 | 51 | Bali | 5101 | Jembrana | 11.66 | 9.83 | 13.73 | 2.26 | 1.46 | 3.32 | 9.20 | 1.06 | 112.60 | 45.80 | 13.90 | 1.94 | 27.45 | 9.01 |
| 271 | | | 5102 | Tabanan | 14.33 | 12.50 | 16.35 | 3.62 | 2.72 | 4.72 | 31.90 | 1.31 | 136.10 | 157.10 | 10.76 | 2.14 | 34.38 | 7.04 |
| 272 | | | 5103 | Badung | 12.24 | 10.55 | 14.11 | 2.89 | 2.09 | 3.88 | 27.50 | 0.66 | 66.40 | 111.50 | 12.23 | 2.66 | 37.41 | 7.46 |
| 273 | | | 5104 | Gianyar | 1.79 | 1.26 | 2.47 | 1.38 | 0.93 | 1.98 | 7.30 | 0.76 | 82.70 | 238.70 | 12.39 | 8.61 | 33.37 | 24.36 |
| 274 | | | 5105 | Klungkung | 16.26 | 14.11 | 18.63 | 2.53 | 1.71 | 3.60 | 37.30 | 1.46 | 106.90 | 204.10 | 14.94 | 2.58 | 27.36 | 7.68 |
| 275 | | | 5106 | Bangli | 12.09 | 10.33 | 14.06 | 3.96 | 2.95 | 5.20 | 37.20 | 1.54 | 294.20 | 238.20 | 13.74 | 2.66 | 31.73 | 11.30 |
| 276 | | | 5107 | Karang Asem | 7.41 | 6.07 | 8.95 | 1.58 | 1.01 | 2.33 | 17.20 | 0.84 | 33.20 | 42.50 | 20.37 | 3.38 | 43.89 | 8.90 |
| 277 | | | 5108 | Buleleng | 9.55 | 8.09 | 11.20 | 2.67 | 1.95 | 3.56 | 15.90 | 0.89 | 116.70 | 80.10 | 20.30 | 5.77 | 61.44 | 18.97 |
| 278 | | | 5171 | Denpasar | 8.64 | 7.26 | 10.20 | 2.65 | 1.91 | 3.57 | 38.90 | 0.39 | 295.50 | 242.10 | 11.34 | 1.61 | 17.93 | 2.64 |
| 279 | 52 | Nusa Tenggara Barat | 5201 | Lombok Barat | 5.62 | 4.48 | 6.97 | 2.04 | 1.39 | 2.90 | 10.40 | 0.81 | 30.00 | 58.50 | 19.46 | 3.25 | 30.10 | 8.01 |

| | | | | | | | | | | | | | | | | | | |
|---|---|---|---|---|---|---|---|---|---|---|---|---|---|---|---|---|---|---|
| 280 | | | 5202 | Lombok Tengah | 8.61 | 7.13 | 10.28 | 0.91 | 0.50 | 1.50 | 6.90 | 0.83 | 50.70 | 80.40 | 15.78 | 3.46 | 41.79 | 12.99 |
| 281 | | | 5203 | Lombok Timur | 7.07 | 5.82 | 8.51 | 1.68 | 1.11 | 2.44 | 7.50 | 0.75 | 37.00 | 82.70 | 17.10 | 3.54 | 36.76 | 9.19 |
| 282 | | | 5204 | Sumbawa | 6.15 | 4.95 | 7.56 | 1.21 | 0.70 | 1.94 | 19.10 | 1.73 | 32.90 | 117.20 | 21.74 | 5.38 | 88.77 | 10.48 |
| 283 | | | 5205 | Dompu | 8.96 | 7.29 | 10.89 | 3.91 | 2.84 | 5.25 | 8.30 | 1.18 | 77.80 | 111.90 | 14.86 | 3.65 | 61.49 | 10.17 |
| 284 | | | 5206 | Bima | 7.58 | 6.24 | 9.11 | 1.09 | 0.62 | 1.78 | 10.90 | 1.31 | 77.30 | 87.60 | 14.57 | 2.79 | 85.19 | 13.87 |
| 285 | | | 5207 | Sumbawa Barat | 2.97 | 2.02 | 4.21 | 0.91 | 0.43 | 1.69 | 23.30 | 2.25 | 44.10 | 333.00 | 13.48 | 6.73 | 33.57 | 16.29 |
| 286 | | | 5208 | Lombok Utara | 4.28 | 3.15 | 5.69 | 1.89 | 1.16 | 2.89 | 10.00 | 1.15 | 39.20 | 67.00 | 22.34 | 5.40 | 52.39 | 13.54 |
| 287 | | | 5271 | Mataram | 9.31 | 7.76 | 11.08 | 1.87 | 1.21 | 2.75 | 44.20 | 0.78 | 270.30 | 254.20 | 10.98 | 3.86 | 14.54 | 5.64 |
| 288 | | | 5272 | Bima | 5.75 | 4.40 | 7.38 | 2.87 | 1.96 | 4.06 | 20.10 | 1.31 | 100.70 | 147.00 | 24.09 | 4.17 | 19.67 | 4.55 |
| 289 | 53 | Nusa Tenggara Timur | 5301 | Sumba Barat | 13.87 | 11.65 | 16.39 | 10.95 | 8.94 | 13.26 | 23.30 | 1.75 | 360.30 | 179.80 | 36.82 | 5.81 | 62.63 | 9.13 |
| 290 | | | 5302 | Sumba Timur | 9.74 | 7.98 | 11.77 | 5.93 | 4.61 | 7.52 | 17.00 | 2.68 | 149.30 | 133.00 | 45.85 | 5.45 | 76.18 | 11.81 |
| 291 | | | 5303 | Kupang | 19.11 | 16.81 | 21.64 | 0.85 | 0.44 | 1.49 | 21.80 | 2.37 | 166.80 | 131.90 | 38.20 | 4.30 | 70.65 | 16.83 |
| 292 | | | 5304 | Timor Tengah Selatan | 8.44 | 6.91 | 10.20 | 1.05 | 0.55 | 1.80 | 10.50 | 1.88 | 20.50 | 62.40 | 38.50 | 4.84 | 46.95 | 9.00 |
| 293 | | | 5305 | Timor Tengah Utara | 5.00 | 3.75 | 6.52 | 2.33 | 1.49 | 3.46 | 4.00 | 3.14 | 70.70 | 170.80 | 18.40 | 3.96 | 41.73 | 5.88 |
| 294 | | | 5306 | Belu | 11.91 | 10.24 | 13.76 | 0.73 | 0.37 | 1.29 | 15.20 | 2.67 | 120.10 | 176.70 | 38.18 | 6.32 | 39.49 | 8.85 |
| 295 | | | 5307 | Alor | 7.03 | 5.52 | 8.82 | 0.64 | 0.25 | 1.32 | 18.00 | 3.50 | 82.10 | 133.20 | 23.05 | 2.54 | 74.36 | 5.97 |
| 296 | | | 5308 | Lembata | 10.42 | 8.49 | 12.66 | 2.07 | 1.26 | 3.21 | 14.10 | 2.12 | 114.50 | 235.20 | 25.06 | 4.97 | 90.85 | 9.78 |
| 297 | | | 5309 | Flores Timur | 7.00 | 5.56 | 8.69 | 1.42 | 0.83 | 2.26 | 13.90 | 2.38 | 68.30 | 146.50 | 24.18 | 3.96 | 37.41 | 16.75 |
| 298 | | | 5310 | Sikka | 10.02 | 8.48 | 11.75 | 2.79 | 2.03 | 3.74 | 10.50 | 2.12 | 101.80 | 117.80 | 22.35 | 3.45 | 18.53 | 2.27 |
| 299 | | | 5311 | Ende | 10.48 | 8.70 | 12.51 | 2.40 | 1.60 | 3.44 | 12.80 | 2.55 | 78.00 | 108.50 | 17.02 | 3.36 | 26.88 | 4.08 |
| 300 | | | 5312 | Ngada | 7.43 | 5.62 | 9.61 | 1.86 | 1.10 | 2.93 | 16.20 | 2.72 | 86.30 | 147.30 | 35.99 | 6.33 | 53.92 | 53.47 |
| 301 | | | 5313 | Manggarai | 6.77 | 5.39 | 8.39 | 1.66 | 0.99 | 2.59 | 5.40 | 1.99 | 86.50 | 108.30 | 19.30 | 3.46 | 48.88 | 5.38 |
| 302 | | | 5314 | Rote Ndao | 13.09 | 10.91 | 15.57 | 1.00 | 0.47 | 1.82 | 22.30 | 2.77 | 0.00 | 97.10 | 35.18 | 5.75 | 59.41 | 22.77 |
| 303 | | | 5315 | Manggarai Barat | 7.21 | 5.68 | 9.03 | 0.24 | 0.05 | 0.72 | 11.20 | 1.87 | 0.00 | 112.10 | 22.70 | 2.82 | 114.29 | 12.86 |
| 304 | | | 5316 | Sumba Tengah | 24.56 | 21.63 | 27.76 | 4.10 | 2.93 | 5.58 | 26.60 | 3.55 | 54.70 | 149.30 | 69.87 | 11.05 | 45.63 | 12.11 |
| 305 | | | 5317 | Sumba Barat Daya | 6.81 | 5.34 | 8.55 | 5.93 | 4.57 | 7.58 | 6.20 | 0.97 | 0.00 | 86.20 | 31.24 | 3.30 | 56.70 | 6.32 |
| 306 | | | 5318 | Nagekeo | 5.69 | 4.33 | 7.33 | 0.99 | 0.48 | 1.76 | 11.40 | 1.49 | 0.00 | 194.50 | 24.59 | 5.01 | 165.32 | 100.45 |
| 307 | | | 5319 | Manggarai Timur | 3.11 | 2.17 | 4.30 | 0.61 | 0.24 | 1.28 | 11.30 | 2.41 | 0.00 | 55.90 | 47.10 | 3.94 | 277.71 | 39.52 |
| 308 | | | 5320 | Sabu Raijua | 9.74 | 7.67 | 12.17 | 0.30 | 0.03 | 1.08 | 2.50 | 2.28 | 67.10 | 34.20 | 59.02 | 4.74 | 58.10 | 16.56 |
| 309 | | | 5371 | Kota Kupang | 10.43 | 8.72 | 12.37 | 3.13 | 2.24 | 4.25 | 22.80 | 0.82 | 151.60 | 172.50 | 18.64 | 4.28 | 41.16 | 12.32 |
| 310 | 61 | Kalimantan Barat | 6101 | Sambas | 5.48 | 4.31 | 6.87 | 0.61 | 0.27 | 1.17 | 4.90 | 1.58 | 69.60 | 152.60 | 18.83 | 4.11 | 65.38 | 12.52 |
| 311 | | | 6102 | Bengkayang | 5.52 | 4.22 | 7.08 | 1.48 | 0.84 | 2.40 | 16.20 | 2.29 | 102.90 | 267.70 | 23.58 | 7.06 | 79.48 | 42.35 |
| 312 | | | 6103 | Landak | 6.41 | 4.98 | 8.11 | 1.24 | 0.57 | 2.25 | 7.60 | 1.41 | 19.10 | 174.80 | 40.41 | 13.90 | 129.09 | 47.11 |
| 313 | | | 6104 | Pontianak | 10.49 | 8.79 | 12.41 | 2.36 | 1.61 | 3.34 | 8.70 | 1.74 | 47.10 | 142.10 | 20.64 | 6.17 | 39.64 | 18.20 |
| 314 | | | 6105 | Sanggau | 2.45 | 1.65 | 3.49 | 0.85 | 0.42 | 1.54 | 11.40 | 1.28 | 68.60 | 165.00 | 21.65 | 8.85 | 118.10 | 60.80 |
| 315 | | | 6106 | Ketapang | 4.18 | 3.12 | 5.46 | 0.25 | 0.07 | 0.64 | 11.10 | 1.63 | 45.50 | 274.80 | 20.62 | 10.38 | 95.01 | 146.54 |
| 316 | | | 6107 | Sintang | 1.99 | 1.30 | 2.90 | 0.45 | 0.19 | 0.86 | 11.90 | 1.59 | 61.80 | 171.00 | 45.31 | 57.44 | 72.44 | 60.31 |
| 317 | | | 6108 | Kapuas Hulu | 2.30 | 1.49 | 3.38 | 1.03 | 0.48 | 1.88 | 15.20 | 3.00 | 62.70 | 322.50 | 24.30 | 10.28 | 112.88 | 90.13 |
| 318 | | | 6109 | Sekadau | 1.88 | 1.16 | 2.87 | 1.08 | 0.55 | 1.88 | 12.20 | 1.92 | 57.00 | 192.20 | 28.66 | 10.26 | 89.20 | 29.80 |
| 319 | | | 6110 | Melawi | 3.30 | 2.31 | 4.58 | 0.95 | 0.45 | 1.76 | 14.10 | 1.79 | 37.90 | 233.40 | 33.49 | 16.13 | 18.60 | 11.60 |
| 320 | | | 6111 | Kayong Utara | 7.34 | 5.72 | 9.26 | 0.09 | 0.00 | 0.49 | 10.10 | 2.43 | 0.00 | 309.50 | 16.22 | 5.45 | 185.46 | 31.10 |
| 321 | | | 6112 | Kubu Raya | 2.69 | 1.91 | 3.68 | 0.64 | 0.30 | 1.18 | 8.90 | 1.16 | 20.50 | 123.50 | 20.91 | 4.24 | 60.11 | 17.10 |
| 322 | | | 6171 | Kota Pontianak | 4.61 | 3.62 | 5.79 | 0.60 | 0.27 | 1.15 | 26.80 | 1.20 | 358.30 | 237.00 | 19.36 | 4.16 | 34.68 | 8.02 |
| 323 | | | 6172 | Singkawang | 7.49 | 6.03 | 9.19 | 3.04 | 2.14 | 4.18 | 38.90 | 0.78 | 558.00 | 388.90 | 12.28 | 3.07 | 21.48 | 5.20 |
| 324 | 62 | Kalimantan Tengah | 6201 | Kotawaringin Barat | 5.74 | 4.20 | 7.61 | 1.68 | 0.97 | 2.69 | 21.00 | 1.90 | 81.60 | 138.20 | 22.97 | 6.17 | 37.82 | 12.88 |
| 325 | | | 6202 | Kotawaringin Timur | 4.46 | 3.26 | 5.94 | 1.73 | 0.98 | 2.81 | 12.20 | 1.50 | 62.90 | 137.00 | 15.85 | 5.03 | 50.10 | 27.69 |
| 326 | | | 6203 | Kapuas | 4.54 | 3.40 | 5.93 | 1.11 | 0.59 | 1.90 | 14.20 | 2.21 | 41.10 | 135.70 | 17.43 | 4.57 | 42.09 | 23.82 |
| 327 | | | 6204 | Barito Selatan | 3.55 | 2.42 | 5.01 | 1.14 | 0.57 | 2.05 | 20.30 | 2.71 | 118.90 | 276.10 | 9.36 | 4.57 | 36.75 | 12.25 |

| | | | | | | | | | | | | | | | | | | |
|---|---|---|---|---|---|---|---|---|---|---|---|---|---|---|---|---|---|---|
| 328 | | | 6205 | Barito Utara | 3.25 | 2.08 | 4.81 | 1.26 | 0.59 | 2.31 | 23.80 | 3.69 | 70.70 | 246.60 | 7.11 | 6.90 | 57.18 | 72.00 |
| 329 | | | 6206 | Sukamara | 10.48 | 8.11 | 13.29 | 2.02 | 1.06 | 3.46 | 39.50 | 3.12 | 0.00 | 270.10 | 8.69 | 5.30 | 34.00 | 29.71 |
| 330 | | | 6207 | Lamandau | 7.21 | 5.55 | 9.22 | 0.34 | 0.09 | 0.88 | 25.10 | 4.88 | 172.90 | 446.30 | 10.90 | 2.68 | 40.70 | 13.90 |
| 331 | | | 6208 | Seruyan | 4.77 | 3.44 | 6.44 | 1.32 | 0.69 | 2.27 | 15.40 | 2.40 | 62.10 | 156.20 | 17.22 | 5.97 | 53.30 | 20.57 |
| 332 | | | 6209 | Katingan | 8.03 | 6.28 | 10.11 | 2.31 | 1.39 | 3.58 | 11.50 | 3.06 | 70.20 | 186.20 | 20.91 | 8.81 | 80.81 | 52.83 |
| 333 | | | 6210 | Pulang Pisau | 1.66 | 0.93 | 2.74 | 0.67 | 0.24 | 1.46 | 19.40 | 2.57 | 52.10 | 121.30 | 16.03 | 6.42 | 66.72 | 128.06 |
| 334 | | | 6211 | Gunung Mas | 2.52 | 1.48 | 4.01 | 0.72 | 0.23 | 1.69 | 24.10 | 4.33 | 49.10 | 259.00 | 25.97 | 21.94 | 170.89 | 115.77 |
| 335 | | | 6212 | Barito Timur | 4.13 | 2.92 | 5.66 | 1.54 | 0.83 | 2.58 | 28.80 | 3.17 | 76.70 | 282.90 | 10.26 | 7.20 | 28.71 | 63.19 |
| 336 | | | 6213 | Murung Raya | 9.41 | 7.03 | 12.25 | 0.79 | 0.31 | 1.64 | 20.20 | 4.05 | 67.50 | 281.50 | 21.25 | 30.07 | 132.23 | 450.43 |
| 337 | | | 6271 | Palangka Raya | 6.99 | 5.25 | 9.07 | 3.03 | 1.89 | 4.56 | 33.40 | 1.27 | 194.00 | 239.60 | 17.47 | 5.19 | 30.15 | 11.06 |
| 338 | 63 | Kalimantan Selatan | 6301 | Tanah Laut | 5.27 | 4.08 | 6.69 | 3.10 | 2.20 | 4.23 | 16.90 | 1.69 | 43.80 | 173.50 | 14.82 | 3.92 | 50.13 | 14.98 |
| 339 | | | 6302 | Kota Baru | 11.38 | 9.42 | 13.63 | 2.86 | 1.94 | 4.04 | 19.20 | 2.59 | 34.50 | 155.20 | 24.18 | 5.75 | 68.66 | 25.45 |
| 340 | | | 6303 | Banjar | 5.86 | 4.64 | 7.30 | 2.07 | 1.36 | 3.03 | 12.20 | 1.26 | 77.50 | 153.90 | 21.00 | 3.74 | 38.64 | 10.83 |
| 341 | | | 6304 | Barito Kuala | 2.79 | 1.91 | 3.93 | 1.15 | 0.64 | 1.90 | 8.10 | 1.91 | 187.20 | 91.60 | 26.57 | 3.90 | 60.63 | 14.45 |
| 342 | | | 6305 | Tapin | 8.32 | 6.68 | 10.23 | 2.23 | 1.41 | 3.33 | 23.20 | 2.15 | 63.50 | 178.80 | 15.53 | 2.86 | 48.67 | 8.97 |
| 343 | | | 6306 | Hulu Sungai Selatan | 5.67 | 4.34 | 7.28 | 3.17 | 2.20 | 4.44 | 16.60 | 2.75 | 90.70 | 150.00 | 14.24 | 3.20 | 31.81 | 7.58 |
| 344 | | | 6307 | Hulu Sungai Tengah | 4.23 | 3.19 | 5.50 | 2.10 | 1.39 | 3.05 | 9.90 | 2.17 | 84.10 | 101.60 | 11.67 | 3.04 | 21.87 | 4.47 |
| 345 | | | 6308 | Hulu Sungai Utara | 3.30 | 2.29 | 4.58 | 1.56 | 0.92 | 2.48 | 8.00 | 1.73 | 85.00 | 79.70 | 11.90 | 2.46 | 19.45 | 4.54 |
| 346 | | | 6309 | Tabalong | 1.79 | 1.08 | 2.79 | 1.12 | 0.57 | 1.97 | 22.00 | 2.03 | 74.20 | 101.70 | 15.42 | 2.95 | 23.91 | 6.03 |
| 347 | | | 6310 | Tanah Bumbu | 3.04 | 2.16 | 4.16 | 1.55 | 0.94 | 2.40 | 17.60 | 1.45 | 41.20 | 96.10 | 17.63 | 3.99 | 31.99 | 7.76 |
| 348 | | | 6311 | Balangan | 5.54 | 4.11 | 7.30 | 3.83 | 2.54 | 5.50 | 29.70 | 2.72 | 95.60 | 230.80 | 15.15 | 3.53 | 40.96 | 13.84 |
| 349 | | | 6371 | Banjarmasin | 4.94 | 3.71 | 6.43 | 3.07 | 2.23 | 4.13 | 34.80 | 1.16 | 264.10 | 276.10 | 11.94 | 4.40 | 24.63 | 6.96 |
| 350 | | | 6372 | Banjar Baru | 3.82 | 2.77 | 5.13 | 0.43 | 0.14 | 1.01 | 31.60 | 1.11 | 154.10 | 126.30 | 11.51 | 3.52 | 19.23 | 6.99 |
| 351 | 64 | Kalimantan Timur | 6401 | Paser | 4.90 | 3.68 | 6.40 | 1.56 | 0.90 | 2.52 | 15.70 | 1.91 | 79.80 | 101.20 | 25.49 | 10.58 | 61.15 | 51.52 |
| 352 | | | 6402 | Kutai Barat | 5.64 | 4.32 | 7.23 | 3.75 | 2.68 | 5.10 | 25.10 | 3.61 | 117.70 | 240.50 | 14.59 | 8.16 | 34.81 | 35.64 |
| 353 | | | 6403 | Kutai Kartanegara | 13.70 | 11.70 | 15.94 | 1.64 | 1.00 | 2.55 | 18.70 | 1.24 | 48.60 | 100.20 | 21.03 | 9.55 | 60.03 | 39.00 |
| 354 | | | 6404 | Kutai Timur | 10.03 | 8.02 | 12.35 | 0.89 | 0.38 | 1.72 | 24.70 | 1.92 | 179.00 | 127.70 | 29.67 | 13.51 | 97.08 | 202.36 |
| 355 | | | 6405 | Berau | 8.89 | 6.94 | 11.19 | 2.54 | 1.62 | 3.78 | 17.80 | 2.46 | 83.90 | 241.00 | 13.08 | 4.01 | 84.69 | 28.52 |
| 356 | | | 6406 | Malinau | 11.00 | 8.64 | 13.77 | 3.69 | 2.44 | 5.34 | 56.60 | 5.38 | 273.30 | 471.80 | 13.95 | 21.32 | 5.00 | 18.22 |
| 357 | | | 6407 | Bulungan | 9.49 | 7.41 | 11.96 | 1.93 | 1.14 | 3.05 | 29.90 | 2.76 | 11.50 | 246.00 | 21.91 | 9.58 | 61.31 | 65.73 |
| 358 | | | 6408 | Nunukan | 9.05 | 7.18 | 11.24 | 0.86 | 0.39 | 1.63 | 38.60 | 2.21 | 115.90 | 179.00 | 16.09 | 5.17 | 99.12 | 2.70 |
| 359 | | | 6409 | Penajam Paser Utara | 8.12 | 6.29 | 10.29 | 1.21 | 0.60 | 2.18 | 27.80 | 1.99 | 41.70 | 129.90 | 21.18 | 7.15 | 80.78 | 41.46 |
| 360 | | | 6410 | Tana Tidung | 12.74 | 9.73 | 16.33 | 1.76 | 0.80 | 3.31 | 45.40 | 6.81 | 0.00 | 573.60 | 8.32 | 3.71 | 120.00 | 36.00 |
| 361 | | | 6471 | Balikpapan | 5.22 | 3.93 | 6.78 | 3.55 | 2.54 | 4.82 | 26.90 | 1.25 | 249.30 | 184.60 | 13.60 | 5.57 | 30.34 | 13.04 |
| 362 | | | 6472 | Samarinda | 5.85 | 4.55 | 7.40 | 2.87 | 1.96 | 4.05 | 24.10 | 0.85 | 250.20 | 186.30 | 14.98 | 5.73 | 45.00 | 14.14 |
| 363 | | | 6473 | Tarakan | 16.59 | 13.98 | 19.53 | 3.68 | 2.57 | 5.09 | 33.50 | 0.94 | 173.20 | 197.30 | 12.74 | 3.48 | 21.05 | 5.50 |
| 364 | | | 6474 | Bontang | 16.02 | 12.90 | 19.56 | 6.86 | 5.03 | 9.06 | 24.00 | 1.08 | 274.00 | 194.10 | 13.81 | 6.33 | 28.64 | 11.21 |
| 365 | 71 | Sulawesi Utara | 7101 | Bolaang Mongondow | 4.38 | 3.25 | 5.77 | 1.35 | 0.75 | 2.23 | 20.10 | 2.14 | 100.40 | 287.40 | 16.43 | 3.04 | 53.16 | 7.64 |
| 366 | | | 7102 | Minahasa | 8.19 | 6.61 | 10.03 | 2.60 | 1.70 | 3.78 | 23.60 | 1.93 | 173.50 | 117.00 | 13.54 | 2.03 | 33.41 | 8.61 |
| 367 | | | 7103 | Kepulauan Sangihe | 5.58 | 4.27 | 7.16 | 1.94 | 1.19 | 2.97 | 50.60 | 3.63 | 293.90 | 322.70 | 22.36 | 3.59 | 78.79 | 16.39 |
| 368 | | | 7104 | Kepulauan Talaud | 6.00 | 4.51 | 7.82 | 1.60 | 0.89 | 2.63 | 38.80 | 7.19 | 194.10 | 73.10 | 24.93 | 3.01 | 182.18 | 87.70 |
| 369 | | | 7105 | Minahasa Selatan | 3.25 | 2.33 | 4.42 | 0.92 | 0.46 | 1.63 | 38.50 | 2.49 | 97.10 | 120.40 | 15.50 | 2.46 | 43.54 | 5.78 |
| 370 | | | 7106 | Minahasa Utara | 8.04 | 6.40 | 9.96 | 2.69 | 1.84 | 3.79 | 40.90 | 1.66 | 39.30 | 135.70 | 23.15 | 4.44 | 35.75 | 4.69 |
| 371 | | | 7107 | Bolaang Mongondow Utr | 4.86 | 3.54 | 6.49 | 1.56 | 0.90 | 2.50 | 43.10 | 4.45 | 27.00 | 84.90 | 14.67 | 3.17 | 135.00 | 228.84 |
| 372 | | | 7108 | Siau Tagulandang Biaro | 4.64 | 3.37 | 6.19 | 2.20 | 1.28 | 3.45 | 70.20 | 5.82 | 53.80 | 337.50 | 19.95 | 5.61 | 50.50 | 14.39 |
| 373 | | | 7109 | Minahasa Tenggara | 6.37 | 4.92 | 8.11 | 2.89 | 1.93 | 4.15 | 37.90 | 3.42 | 62.60 | 241.00 | 13.69 | 5.18 | 66.80 | 17.91 |
| 374 | | | 7110 | Bolaang Mongondow Slt | 2.82 | 1.79 | 4.21 | 0.48 | 0.13 | 1.24 | 21.70 | 3.51 | 83.60 | 97.00 | 23.82 | 4.62 | 130.96 | 17.23 |
| 375 | | | 7111 | Bolaang Mongondow Tm | 3.07 | 2.07 | 4.40 | 0.59 | 0.21 | 1.29 | 18.00 | 2.25 | 0.00 | 89.80 | 13.66 | 3.47 | 66.61 | 14.51 |

| | | | | | | | | | | | | | | | | | | |
|---|---|---|---|---|---|---|---|---|---|---|---|---|---|---|---|---|---|---|
| 376 | | | 7171 | Manado | 8.79 | 7.31 | 10.49 | 2.72 | 1.90 | 3.76 | 74.50 | 1.11 | 547.60 | 451.00 | 14.04 | 2.03 | 36.39 | 4.46 |
| 377 | | | 7172 | Bitung | 8.28 | 6.64 | 10.20 | 4.47 | 3.28 | 5.94 | 39.60 | 1.37 | 178.20 | 194.00 | 22.82 | 3.69 | 39.68 | 4.30 |
| 378 | | | 7173 | Tomohon | 12.69 | 10.53 | 15.15 | 6.03 | 4.59 | 7.77 | 50.00 | 2.19 | 502.70 | 457.90 | 11.79 | 1.39 | 29.01 | 2.52 |
| 379 | | | 7174 | Kotamobagu | 5.48 | 4.04 | 7.25 | 3.04 | 2.01 | 4.41 | 31.90 | 1.33 | 172.00 | 156.90 | 10.51 | 2.73 | 15.49 | 3.41 |
| 380 | 72 | Sulawesi Tengah | 7201 | Banggai Kepulauan | 8.11 | 6.32 | 10.23 | 0.64 | 0.23 | 1.40 | 9.70 | 2.43 | 41.10 | 288.20 | 19.33 | 3.94 | 50.00 | 4.76 |
| 381 | | | 7202 | Banggai | 4.71 | 3.59 | 6.06 | 2.64 | 1.80 | 3.73 | 20.10 | 2.06 | 76.30 | 246.60 | 15.27 | 3.40 | 26.71 | 4.71 |
| 382 | | | 7203 | Morowali | 4.75 | 3.46 | 6.35 | 3.82 | 2.67 | 5.29 | 17.10 | 2.56 | 49.90 | 226.30 | 24.44 | 4.83 | 49.16 | 16.68 |
| 383 | | | 7204 | Poso | 4.50 | 3.32 | 5.96 | 3.20 | 2.24 | 4.43 | 16.90 | 2.79 | 156.60 | 275.50 | 13.26 | 3.41 | 77.25 | 12.66 |
| 384 | | | 7205 | Donggala | 3.44 | 2.48 | 4.64 | 2.83 | 1.97 | 3.93 | 14.40 | 1.50 | 42.80 | 123.70 | 31.37 | 3.71 | 128.11 | 25.05 |
| 385 | | | 7206 | Toli-Toli | 3.85 | 2.76 | 5.22 | 2.64 | 1.75 | 3.81 | 12.30 | 1.84 | 43.90 | 194.10 | 31.88 | 4.42 | 41.69 | 10.33 |
| 386 | | | 7207 | Buol | 9.89 | 8.07 | 12.00 | 1.75 | 1.06 | 2.71 | 11.20 | 2.31 | 61.70 | 364.70 | 25.24 | 5.20 | 78.55 | 20.59 |
| 387 | | | 7208 | Parigi Moutong | 3.36 | 2.40 | 4.58 | 0.86 | 0.46 | 1.48 | 5.80 | 1.41 | 50.90 | 166.50 | 29.69 | 3.53 | 139.22 | 44.22 |
| 388 | | | 7209 | Tojo Una-Una | 9.43 | 7.53 | 11.66 | 4.87 | 3.55 | 6.51 | 16.80 | 2.63 | 92.90 | 210.80 | 27.57 | 6.01 | 109.04 | 41.42 |
| 389 | | | 7210 | Sigi | 3.89 | 2.84 | 5.18 | 4.66 | 3.51 | 6.04 | 9.90 | 2.46 | 0.00 | 176.50 | 21.10 | 2.35 | 59.02 | 12.89 |
| 390 | | | 7271 | Palu | 9.13 | 7.40 | 11.13 | 5.81 | 4.47 | 7.42 | 56.80 | 0.99 | 485.90 | 411.20 | 11.93 | 3.87 | 20.22 | 5.72 |
| 391 | 73 | Sulawesi Selatan | 7301 | Kepulauan Selayar | 6.08 | 4.46 | 8.08 | 1.70 | 0.98 | 2.74 | 18.80 | 3.29 | 68.20 | 175.60 | 27.27 | 5.65 | 95.15 | 15.95 |
| 392 | | | 7302 | Bulukumba | 13.30 | 11.38 | 15.45 | 1.37 | 0.81 | 2.16 | 12.60 | 1.38 | 63.10 | 94.60 | 23.82 | 3.50 | 61.44 | 8.58 |
| 393 | | | 7303 | Bantaeng | 9.64 | 7.83 | 11.72 | 3.11 | 2.14 | 4.36 | 16.20 | 2.11 | 59.60 | 193.90 | 13.93 | 2.09 | 24.01 | 3.88 |
| 394 | | | 7304 | Jeneponto | 18.58 | 16.22 | 21.18 | 5.12 | 3.90 | 6.59 | 8.90 | 1.51 | 26.20 | 99.40 | 17.51 | 2.82 | 44.35 | 6.39 |
| 395 | | | 7305 | Takalar | 11.05 | 9.26 | 13.08 | 3.94 | 2.90 | 5.22 | 12.10 | 1.60 | 94.40 | 170.90 | 16.26 | 2.66 | 36.03 | 7.03 |
| 396 | | | 7306 | Gowa | 11.72 | 10.11 | 13.51 | 4.19 | 3.25 | 5.31 | 10.40 | 1.10 | 42.50 | 50.90 | 24.93 | 2.76 | 60.88 | 8.93 |
| 397 | | | 7307 | Sinjai | 6.99 | 5.56 | 8.67 | 1.43 | 0.82 | 2.31 | 20.10 | 2.01 | 63.10 | 164.30 | 17.50 | 2.05 | 30.00 | 1.62 |
| 398 | | | 7308 | Maros | 6.14 | 4.61 | 7.99 | 0.57 | 0.17 | 1.37 | 16.80 | 1.26 | 83.40 | 162.30 | 24.29 | 3.06 | 49.26 | 7.14 |
| 399 | | | 7309 | Pangkajene dan Kep | 7.86 | 6.39 | 9.56 | 2.07 | 1.35 | 3.02 | 19.70 | 2.16 | 74.50 | 129.90 | 22.36 | 4.10 | 91.25 | 30.29 |
| 400 | | | 7310 | Barru | 11.57 | 9.74 | 13.65 | 2.56 | 1.72 | 3.65 | 19.00 | 2.68 | 85.90 | 173.50 | 15.55 | 2.31 | 37.64 | 5.69 |
| 401 | | | 7311 | Bone | 2.92 | 2.16 | 3.86 | 0.90 | 0.52 | 1.46 | 9.10 | 1.52 | 53.30 | 94.10 | 23.49 | 2.48 | 32.49 | 4.34 |
| 402 | | | 7312 | Soppeng | 6.15 | 4.85 | 7.67 | 2.08 | 1.31 | 3.13 | 4.70 | 2.18 | 64.10 | 139.40 | 14.51 | 2.31 | 47.71 | 9.77 |
| 403 | | | 7313 | Wajo | 7.97 | 6.63 | 9.51 | 2.17 | 1.49 | 3.06 | 11.70 | 1.71 | 68.30 | 130.70 | 13.00 | 1.73 | 55.00 | 6.96 |
| 404 | | | 7314 | Sidenreng Rappang | 10.15 | 8.53 | 11.99 | 2.23 | 1.50 | 3.18 | 15.50 | 1.48 | 94.30 | 144.30 | 13.30 | 2.15 | 25.88 | 3.90 |
| 405 | | | 7315 | Pinrang | 8.32 | 6.81 | 10.07 | 4.08 | 3.03 | 5.38 | 14.40 | 1.23 | 74.10 | 114.20 | 29.85 | 8.70 | 32.02 | 9.10 |
| 406 | | | 7316 | Enrekang | 9.63 | 8.01 | 11.46 | 3.03 | 2.14 | 4.16 | 12.60 | 1.96 | 96.60 | 140.80 | 15.26 | 1.91 | 70.86 | 13.86 |
| 407 | | | 7317 | Luwu | 5.62 | 4.40 | 7.07 | 3.98 | 2.97 | 5.23 | 13.50 | 1.81 | 140.50 | 107.90 | 23.50 | 3.14 | 44.03 | 7.01 |
| 408 | | | 7318 | Tana Toraja | 9.17 | 7.31 | 11.36 | 2.26 | 1.37 | 3.49 | 19.50 | 2.73 | 262.80 | 132.00 | 53.19 | 3.36 | 96.36 | 24.56 |
| 409 | | | 7322 | Luwu Utara | 11.81 | 9.85 | 14.04 | 2.19 | 1.41 | 3.26 | 12.00 | 1.30 | 0.00 | 108.50 | 21.57 | 3.69 | 22.50 | 9.18 |
| 410 | | | 7325 | Luwu Timur | 8.15 | 6.48 | 10.12 | 1.28 | 0.68 | 2.17 | 14.20 | 1.77 | 65.80 | 126.40 | 16.39 | 3.65 | 12.59 | 7.88 |
| 411 | | | 7326 | Toraja Utara | 10.67 | 8.81 | 12.81 | 6.83 | 5.37 | 8.55 | 15.50 | 3.31 | 0.00 | 126.30 | 30.85 | 4.54 | 41.76 | 20.23 |
| 412 | | | 7371 | Makassar | 6.35 | 5.19 | 7.70 | 4.97 | 3.95 | 6.16 | 32.50 | 0.92 | 431.90 | 223.60 | 16.35 | 3.66 | 26.87 | 6.34 |
| 413 | | | 7372 | Parepare | 11.58 | 9.68 | 13.75 | 3.00 | 2.07 | 4.21 | 30.40 | 1.33 | 393.90 | 454.60 | 21.23 | 2.06 | 26.96 | 2.80 |
| 414 | | | 7373 | Palopo | 9.60 | 7.77 | 11.73 | 4.98 | 3.65 | 6.63 | 27.80 | 2.13 | 330.60 | 351.90 | 18.98 | 3.20 | 23.36 | 4.92 |
| 415 | 74 | Sulawesi Tenggara | 7401 | Buton | 2.37 | 1.47 | 3.61 | 0.44 | 0.12 | 1.15 | 5.80 | 3.57 | 21.30 | 188.50 | 19.93 | 3.85 | 89.74 | 19.57 |
| 416 | | | 7402 | Muna | 2.44 | 1.60 | 3.55 | 0.56 | 0.20 | 1.23 | 23.10 | 4.34 | 31.70 | 73.30 | 12.98 | 3.04 | 39.59 | 9.96 |
| 417 | | | 7403 | Konawe | 8.82 | 7.22 | 10.66 | 4.35 | 3.24 | 5.72 | 17.60 | 3.55 | 0.00 | 122.90 | 17.27 | 3.27 | 18.63 | 9.87 |
| 418 | | | 7404 | Kolaka | 19.06 | 16.59 | 21.77 | 2.73 | 1.85 | 3.87 | 13.20 | 2.20 | 90.30 | 160.80 | 15.95 | 3.83 | 56.50 | 14.41 |
| 419 | | | 7405 | Konawe Selatan | 6.37 | 5.03 | 7.94 | 1.92 | 1.22 | 2.86 | 12.20 | 2.41 | 17.50 | 78.20 | 27.84 | 7.81 | 85.86 | 55.37 |
| 420 | | | 7406 | Bombana | 5.60 | 4.15 | 7.37 | 0.63 | 0.25 | 1.30 | 24.50 | 4.38 | 23.20 | 112.70 | 17.97 | 3.62 | 49.51 | 12.23 |
| 421 | | | 7407 | Wakatobi | 9.15 | 7.19 | 11.47 | 0.46 | 0.08 | 1.37 | 14.90 | 5.96 | 76.50 | 255.20 | 13.57 | 2.38 | 119.45 | 47.61 |
| 422 | | | 7408 | Kolaka Utara | 13.48 | 11.35 | 15.88 | 0.75 | 0.34 | 1.43 | 13.70 | 3.65 | 76.10 | 126.30 | 16.88 | 4.10 | 152.02 | 23.65 |
| 423 | | | 7409 | Buton Utara | 5.21 | 3.82 | 6.93 | 0.21 | 0.02 | 0.77 | 32.10 | 5.06 | 32.10 | 325.60 | 9.11 | 2.11 | 60.25 | 9.53 |

| | | | | | | | | | | | | | | | | | |
|---|---|---|---|---|---|---|---|---|---|---|---|---|---|---|---|---|---|
| 424 | | | 7410 | Konawe Utara | 4.10 | 2.83 | 5.74 | 1.20 | 0.57 | 2.20 | 12.50 | 7.63 | 86.00 | 96.80 | 32.43 | 5.82 | 89.61 | 31.93 |
| 425 | | | 7411 | Kendari | 6.64 | 5.24 | 8.29 | 1.77 | 1.07 | 2.72 | 32.50 | 1.43 | 278.30 | 259.80 | 22.62 | 2.90 | 38.08 | 5.52 |
| 426 | | | 7412 | Baubau | 8.37 | 6.64 | 10.42 | 0.71 | 0.25 | 1.55 | 25.60 | 3.44 | 89.60 | 212.30 | 10.09 | 2.03 | 19.42 | 3.96 |
| 427 | 75 | Gorontalo | 7501 | Boalemo | 9.14 | 7.34 | 11.24 | 2.84 | 1.90 | 4.09 | 22.70 | 2.34 | 61.70 | 120.60 | 18.56 | 2.87 | 38.12 | 11.11 |
| 428 | | | 7502 | Gorontalo | 8.82 | 7.30 | 10.57 | 2.08 | 1.37 | 3.01 | 33.70 | 1.62 | 99.20 | 192.40 | 17.09 | 1.95 | 44.21 | 5.75 |
| 429 | | | 7503 | Pohuwato | 5.45 | 4.14 | 7.03 | 3.53 | 2.51 | 4.83 | 20.70 | 3.42 | 84.00 | 151.00 | 13.77 | 2.08 | 73.75 | 5.60 |
| 430 | | | 7504 | Bone Bolango | 8.52 | 6.80 | 10.53 | 2.36 | 1.55 | 3.44 | 22.00 | 3.88 | 129.90 | 111.10 | 10.72 | 2.39 | 38.23 | 6.15 |
| 431 | | | 7505 | Gorontalo Utara | 13.08 | 10.78 | 15.71 | 1.62 | 0.90 | 2.68 | 26.40 | 3.96 | 10.60 | 154.10 | 19.42 | 2.14 | 155.75 | 20.81 |
| 432 | | | 7571 | Kota Gorontalo | 18.55 | 16.32 | 20.99 | 6.11 | 4.86 | 7.58 | 9.20 | 1.53 | 277.90 | 62.10 | 17.66 | 1.75 | 18.13 | 3.49 |
| 433 | 76 | Sulawesi Barat | 7601 | Majene | 10.87 | 8.90 | 13.16 | 2.59 | 1.67 | 3.82 | 12.50 | 1.97 | 77.60 | 214.90 | 16.09 | 2.51 | 48.23 | 6.00 |
| 434 | | | 7602 | Polewali Mandar | 3.28 | 2.39 | 4.41 | 1.45 | 0.88 | 2.25 | 9.80 | 1.37 | 55.10 | 81.50 | 21.49 | 3.33 | 24.89 | 4.01 |
| 435 | | | 7603 | Mamasa | 8.71 | 6.97 | 10.75 | 0.58 | 0.18 | 1.36 | 18.70 | 3.28 | 105.60 | 139.10 | 34.91 | 13.32 | 43.91 | 9.17 |
| 436 | | | 7604 | Mamuju | 1.75 | 1.07 | 2.69 | 1.23 | 0.68 | 2.05 | 27.00 | 2.57 | 34.80 | 132.20 | 16.82 | 4.66 | 68.09 | 14.24 |
| 437 | | | 7605 | Mamuju Utara | 4.44 | 3.04 | 6.22 | 1.66 | 0.95 | 2.67 | 20.10 | 2.82 | 43.00 | 126.20 | 21.21 | 3.09 | 17.96 | 3.60 |
| 438 | 81 | Maluku | 8101 | Maluku Tenggara | 9.43 | 7.57 | 11.60 | 1.32 | 0.71 | 2.23 | 26.10 | 4.19 | 265.80 | 576.20 | 12.99 | 2.34 | 50.30 | 12.44 |
| 439 | | | 8102 | Maluku Tenggara Barat | 14.30 | 12.00 | 16.91 | 4.13 | 2.93 | 5.63 | 15.30 | 3.32 | 55.40 | 260.80 | 17.16 | 6.28 | 87.07 | 58.58 |
| 440 | | | 8103 | Maluku Tengah | 7.11 | 5.76 | 8.68 | 1.26 | 0.73 | 2.02 | 28.30 | 2.46 | 69.90 | 200.80 | 70.78 | 7.27 | 149.06 | 44.45 |
| 441 | | | 8104 | Buru | 3.31 | 2.20 | 4.77 | 1.24 | 0.60 | 2.25 | 16.60 | 2.24 | 45.20 | 337.80 | 38.90 | 13.90 | 76.80 | 319.64 |
| 442 | | | 8105 | Kepulauan Aru | 5.99 | 4.48 | 7.83 | 0.87 | 0.37 | 1.74 | 27.70 | 7.68 | 131.80 | 465.20 | 43.71 | 14.13 | 177.61 | 56.28 |
| 443 | | | 8106 | Seram Bagian Barat | 1.91 | 1.14 | 2.99 | 0.18 | 0.02 | 0.65 | 12.00 | 2.78 | 26.30 | 278.60 | 25.45 | 7.74 | 113.20 | 26.00 |
| 444 | | | 8107 | Seram Bagian Timur | 3.20 | 2.08 | 4.69 | 0.10 | 0.00 | 0.54 | 19.90 | 5.17 | 70.40 | 346.20 | 36.79 | 32.90 | 381.83 | 1329.05 |
| 445 | | | 8108 | Maluku Barat Daya | 9.72 | 7.81 | 11.95 | 0.29 | 0.06 | 0.83 | 24.10 | 7.62 | 0.00 | 243.80 | 33.83 | 17.63 | 490.91 | 1560.00 |
| 446 | | | 8109 | Buru Selatan | 2.16 | 1.04 | 3.85 | 0.56 | 0.01 | 2.21 | 18.40 | 6.02 | 14.30 | 162.20 | 28.07 | 7.39 | 54.30 | 51.88 |
| 447 | | | 8171 | Ambon | 7.83 | 6.38 | 9.51 | 2.42 | 1.65 | 3.43 | 28.20 | 6.03 | 166.10 | 299.20 | 21.01 | 4.26 | 47.32 | 8.83 |
| 448 | | | 8172 | Tual | 11.01 | 8.88 | 13.48 | 3.79 | 2.55 | 5.40 | 13.90 | 1.79 | 0.00 | 326.10 | 16.12 | 3.86 | 41.12 | 38.82 |
| 449 | 82 | Maluku Utara | 8201 | Halmahera Barat | 4.72 | 3.42 | 6.35 | 2.21 | 1.36 | 3.39 | 26.30 | 2.99 | 272.60 | 247.20 | 23.14 | 8.19 | 64.84 | 49.51 |
| 450 | | | 8202 | Halmahera Tengah | 13.88 | 10.93 | 17.36 | 0.55 | 0.06 | 2.01 | 42.50 | 7.01 | 288.90 | 533.10 | 10.26 | 5.55 | 50.00 | 172.41 |
| 451 | | | 8203 | Kepulauan Sula | 3.52 | 2.33 | 5.07 | 0.52 | 0.17 | 1.21 | 19.90 | 3.50 | 58.30 | 245.70 | 13.32 | 6.17 | 79.38 | 8.24 |
| 452 | | | 8204 | Halmahera Selatan | 6.31 | 4.85 | 8.06 | 2.42 | 1.57 | 3.56 | 18.30 | 4.11 | 52.10 | 264.70 | 9.23 | 5.08 | 30.00 | 32.50 |
| 453 | | | 8205 | Halmahera Utara | 7.01 | 5.47 | 8.84 | 1.44 | 0.82 | 2.34 | 19.10 | 2.87 | 88.80 | 225.30 | 21.70 | 3.87 | 63.69 | 13.54 |
| 454 | | | 8206 | Halmahera Timur | 8.11 | 6.35 | 10.19 | 0.72 | 0.28 | 1.49 | 28.60 | 5.22 | 128.10 | 439.10 | 28.94 | 6.54 | 138.85 | 81.17 |
| 455 | | | 8207 | Pulau Morotai | 9.20 | 7.15 | 11.65 | 1.93 | 1.03 | 3.27 | 22.40 | 3.11 | 27.60 | 253.70 | 17.75 | 12.46 | 59.05 | 65.49 |
| 456 | | | 8271 | Ternate | 11.27 | 9.25 | 13.56 | 4.79 | 3.61 | 6.22 | 29.40 | 1.47 | 256.10 | 257.60 | 24.27 | 5.96 | 32.93 | 11.62 |
| 457 | | | 8272 | Tidore Kepulauan | 7.79 | 6.14 | 9.76 | 2.83 | 1.89 | 4.07 | 23.20 | 3.03 | 0.00 | 237.30 | 10.43 | 3.21 | 41.22 | 18.97 |
| 458 | 91 | Papua Barat | 9101 | Fakfak | 17.45 | 14.57 | 20.70 | 4.54 | 3.05 | 6.45 | 33.70 | 3.89 | 265.80 | 378.70 | 16.13 | 8.02 | 35.25 | 13.93 |
| 459 | | | 9102 | Kaimana | 22.16 | 18.16 | 26.72 | 2.87 | 1.54 | 4.78 | 30.00 | 4.50 | 674.60 | 481.60 | 11.71 | 9.20 | 24.68 | 305.81 |
| 460 | | | 9103 | Teluk Wondama | 7.87 | 5.58 | 10.74 | 0.89 | 0.29 | 2.01 | 59.30 | 5.93 | 493.90 | 500.50 | 19.32 | 12.08 | 89.23 | 936.20 |
| 461 | | | 9104 | Teluk Bintuni | 3.61 | 2.07 | 5.72 | 0.12 | 0.00 | 0.67 | 19.80 | 9.92 | 114.10 | 52.90 | 41.76 | 10.73 | 120.00 | 180.00 |
| 462 | | | 9105 | Manokwari | 6.09 | 4.37 | 8.18 | 3.00 | 1.63 | 4.85 | 14.30 | 3.74 | 0.00 | 227.10 | 13.79 | 7.96 | 14.94 | 9.89 |
| 463 | | | 9106 | Sorong Selatan | 12.34 | 9.01 | 16.46 | 1.04 | 0.26 | 2.69 | 25.10 | 10.25 | 300.70 | 268.80 | 15.00 | 4.50 | 26.82 | 21.68 |
| 464 | | | 9107 | Sorong | 10.91 | 8.58 | 13.63 | 0.61 | 0.22 | 1.33 | 68.70 | 6.26 | 0.00 | 630.80 | 23.79 | 7.97 | 113.18 | 77.10 |
| 465 | | | 9108 | Raja Ampat | 12.30 | 9.52 | 15.61 | 1.06 | 0.42 | 2.20 | 28.50 | 11.62 | 155.00 | 628.00 | 9.33 | 1.90 | 75.84 | 51.75 |
| 466 | | | 9109 | Tambrau | 17.83 | 13.16 | 23.56 | 0.85 | 0.17 | 2.50 | 113.80 | 21.34 | 0.00 | 441.10 | 220.00 | 2.07 | 455.84 | 284.49 |
| 467 | | | 9110 | Maybrat | 15.34 | 12 | 19.3 | 1.39 | 0.55 | 2.88 | 18.40 | 11.03 | 0.00 | 404.50 | 25.82 | 32.34 | 192.50 | 220.50 |
| 468 | | | 9171 | Kota Sorong | 9.77 | 7.6 | 12.3 | 3.60 | 2.38 | 5.18 | 43.20 | 0.82 | 258.20 | 163.70 | 13.30 | 5.14 | 16.26 | 6.71 |
| 469 | 94 | Papua | 9401 | Merauke | 9.33 | 7.41 | 11.6 | 1.69 | 0.92 | 2.8 | 20.30 | 2.49 | 169.00 | 170.70 | 25.35 | 15.13 | 42.67 | 60.72 |
| 470 | | | 9402 | Jayawijaya | 8.18 | 6.06 | 10.7 | 0.19 | 0.02 | 0.67 | 9.90 | 1.62 | 301.70 | 146.30 | 71.29 | 14.86 | 21.76 | 30.40 |
| 471 | | | 9403 | Jayapura | 8.76 | 6.84 | 11 | 1.53 | 0.77 | 2.71 | 5.10 | 4.14 | 143.00 | 116.20 | 20.62 | 8.79 | 76.12 | 46.07 |

| | | | | | | | | | | | | | | | | | | |
|---|---|---|---|---|---|---|---|---|---|---|---|---|---|---|---|---|---|---|
| 472 | | | 9404 | Nabire | 8.15 | 6.35 | 10.3 | 1.78 | 1.02 | 2.86 | 18.80 | 4.88 | 42.50 | 232.70 | 27.48 | 4.30 | 15.67 | 5.27 |
| 473 | | | 9408 | Kepulauan Yapen | 19.81 | 16.9 | 23.1 | 4.47 | 3.15 | 6.15 | 25.50 | 3.82 | 0.00 | 346.80 | 22.02 | 8.83 | 23.33 | 4.75 |
| 474 | | | 9409 | Biak Numfor | 18.39 | 15.9 | 21.2 | 2.50 | 1.63 | 3.68 | 26.30 | 3.46 | 283.90 | 222.40 | 28.84 | 6.28 | 72.63 | 26.94 |
| 475 | | | 9410 | Paniai | 25.36 | 19.7 | 31.7 | 10.15 | 7.54 | 13.3 | 7.40 | 2.86 | 0.00 | 91.10 | 48.45 | 5.45 | 130.91 | 58.40 |
| 476 | | | 9411 | Puncak Jaya | 48.53 | 39.9 | 57.9 | 1.33 | 0.21 | 4.62 | 20.90 | 1.93 | 74.20 | 136.60 | 70.04 | 11.38 | 225.36 | 201.45 |
| 477 | | | 9412 | Mimika | 15.01 | 12.1 | 18.3 | 2.77 | 1.14 | 5.06 | 25.90 | 1.74 | 83.90 | 230.00 | 16.46 | 5.21 | 68.46 | 1202.58 |
| 478 | | | 9413 | Boeven Digoel | 19.93 | 15.5 | 25 | 3.06 | 1.73 | 4.94 | 23.30 | 8.74 | 97.60 | 278.30 | 19.69 | 14.69 | 35.29 | 126.98 |
| 479 | | | 9414 | Mappi | 7.20 | 4.42 | 10.6 | 0.44 | 0.11 | 1.16 | 23.90 | 3.28 | 43.80 | 163.20 | 24.94 | 14.31 | 142.04 | 703.70 |
| 480 | | | 9415 | Asmat | 5.19 | 2.53 | 8.72 | 0.39 | 0.03 | 1.45 | 30.80 | 4.14 | 81.70 | 200.60 | 57.07 | 33.78 | 111.50 | 1434.79 |
| 481 | | | 9416 | Yahukimo | 12.01 | 9.61 | 14.8 | 0.57 | 0.22 | 1.19 | 21.70 | 4.59 | 0.00 | 72.60 | 37.00 | 12.22 | 97.91 | 148.00 |
| 482 | | | 9417 | Pegunungan Bintang | 22.02 | 17.6 | 27 | 1.01 | 0.48 | 1.88 | 29.80 | 10.81 | 119.20 | 73.30 | 5.00 | 20.53 | 48.15 | 399.85 |
| 483 | | | 9418 | Tolikara | 9.13 | 6.54 | 12.3 | 0.00 | - | - | 12.80 | 5.33 | 0.00 | 94.50 | 2.00 | 1.00 | 60.00 | 203.68 |
| 484 | | | 9419 | Sarmi | 5.65 | 3.97 | 7.79 | 0.00 | - | - | 61.60 | 6.66 | 0.00 | 170.10 | 30.96 | 35.23 | 358.72 | 532.92 |
| 485 | | | 9420 | Keerom | 18.32 | 15.3 | 21.8 | 5.06 | 3.47 | 7.12 | 43.50 | 5.02 | 182.50 | 98.80 | 15.74 | 4.70 | 121.14 | 74.37 |
| 486 | | | 9426 | Waropen | 5.10 | 3.18 | 7.76 | 0.00 | - | - | 6.60 | 9.90 | 0.00 | 455.20 | 42.82 | 10.83 | 255.00 | 629.00 |
| 487 | | | 9427 | Supiori | 4.36 | 2.77 | 6.48 | 0.74 | 0.24 | 1.74 | 71.70 | 7.68 | 256.00 | 481.20 | 5.00 | 3.87 | 45.00 | 190.25 |
| 488 | | | 9428 | Mamberamo Raya | 8.81 | 5.92 | 12.5 | 1.09 | 0.21 | 3.24 | 84.10 | 9.29 | 79.70 | 446.90 | 153.79 | 50.81 | 369.66 | 3277.37 |
| 489 | | | 9429 | Nduga | 4.09 | 2.27 | 6.56 | 0.99 | 0.09 | 3.15 | 17.50 | 2.47 | 0.00 | 39.10 | 97.50 | 0.00 | 35.00 | 370.00 |
| 490 | | | 9430 | Lanny Jaya | 13.91 | 10.3 | 18.1 | 0.70 | 0.04 | 2.48 | 4.40 | 1.64 | 13.10 | 62.90 | 111.74 | 32.37 | 154.00 | 127.08 |
| 491 | | | 9431 | Mamberamo Tengah | 8.96 | 4 | 15.2 | 0.00 | - | - | 22.60 | 3.08 | 0.00 | 94.60 | 86.99 | 3.52 | 150.00 | 51.80 |
| 492 | | | 9432 | Yalimo | 6.44 | 4.59 | 8.71 | 0.37 | 0.03 | 1.36 | 16.00 | 3.36 | 0.00 | 17.60 | 68.80 | 11.79 | 178.90 | 568.07 |
| 493 | | | 9433 | Puncak | 26.16 | 22 | 30.8 | 0.21 | 0.02 | 0.77 | 10.50 | 2.09 | 0.00 | 21.80 | 40.00 | 0.00 | 142.73 | 444.00 |
| 494 | | | 9434 | Dogiyai | 9.47 | 6.87 | 12.6 | 0.00 | - | - | 6.80 | 2.89 | 0.00 | 68.50 | 58.29 | 20.41 | 453.28 | 169.04 |
| 495 | | | 9435 | Intan Jaya | 59.37 | 47.8 | 72.2 | 0.00 | - | - | 8.00 | 3.61 | 0.00 | 166.60 | 302.30 | 0.00 | 331.30 | 908.91 |
| 496 | | | 9436 | Deiyai | 9.63 | 6.91 | 13 | 0.65 | 0.18 | 1.68 | 3.90 | 3.92 | 0.00 | 81.10 | 58.69 | 0.00 | 51.88 | 50.88 |
| 497 | | | 9471 | Kota Jayapura | 4.64 | 3.32 | 6.29 | 0.76 | 0.3 | 1.57 | 47.50 | 1.14 | 370.70 | 271.60 | 32.87 | 5.24 | 56.14 | 91.34 |
| | | | | | | | | | | | | | | | | | | |
